# Efficient exploration of pan-cancer networks by generalized covariance selection and interactive web content

**DOI:** 10.1093/nar/gkv413

**Published:** 2015-05-07

**Authors:** Teresia Kling, Patrik Johansson, José Sanchez, Voichita D. Marinescu, Rebecka Jörnsten, Sven Nelander

**Affiliations:** 1 Sahlgrenska Cancer Center and Dept of Molecular and Clinical Medicine, University of Gothenburg, Box 425, SE-405 30 Gothenburg, Sweden; 2 Department of Immunology, Genetics and Pathology (IGP) and Science for Life Laboratory, Uppsala University, Rudbecklaboratoriet, SE-751 85 Uppsala, Sweden; 3 Mathematical Sciences, University of Gothenburg and Chalmers University of Technology, SE-412 96 Gothenburg, Sweden

## Abstract

Statistical network modeling techniques are increasingly important tools to analyze cancer genomics data. However, current tools and resources are not designed to work across multiple diagnoses and technical platforms, thus limiting their applicability to comprehensive pan-cancer datasets such as The Cancer Genome Atlas (TCGA). To address this, we describe a new data driven modeling method, based on generalized Sparse Inverse Covariance Selection (SICS). The method integrates genetic, epigenetic and transcriptional data from multiple cancers, to define links that are present in multiple cancers, a subset of cancers, or a single cancer. It is shown to be statistically robust and effective at detecting direct pathway links in data from TCGA. To facilitate interpretation of the results, we introduce a publicly accessible tool (cancerlandscapes.org), in which the derived networks are explored as interactive web content, linked to several pathway and pharmacological databases. To evaluate the performance of the method, we constructed a model for eight TCGA cancers, using data from 3900 patients. The model rediscovered known mechanisms and contained interesting predictions. Possible applications include prediction of regulatory relationships, comparison of network modules across multiple forms of cancer and identification of drug targets.

## INTRODUCTION

Advances in molecular profiling of cancer motivate the development of computational tools to access and interpret the data. For instance, one important goal of cancer systems biology is to understand how genetic lesions drive the phenotype of cancer cells and contribute to disease progression (1,2). Another increasingly important challenge is to integrate molecular data from several different cancers to identify common vulnerabilities that can be exploited therapeutically (3,4). To achieve such aims, effective data-driven modeling strategies will be important, if not essential. We have developed a novel tool for robust statistical network analysis of multidimensional cancer genome data across multiple diagnoses. The key components of this approach are (i) integration of data from multiple cancers and data types, (ii) network model construction by means of statistical optimization, (iii) statistical functional assessment of modules in the resulting network; and, (iv) visualization of the results as interactive web content. The method relies on an efficient estimation algorithm and is designed to integrate five types of cancer genome data: DNA point mutations, DNA methylation profiles, DNA copy number aberration profiles, and mRNA and miRNA transcriptional measurements.

### Network models of cancer

In the context of genome-scale data analysis, statistical network modeling is a broad family of methods that seek to describe variation in the data in terms of a network of pairwise variable couplings (1,2). Examples of such statistical methods include WGCNA (5), which constructs a network from Pearson correlations, and the information theory based ARACNE (6). Application of network modeling to mRNA data from cancers resulted in pioneering discoveries, including the identification of regulators of epithelial-mesenchymal transitions in brain tumors (7) and the key regulators in B-cell lymphoma (8). Current cancer genomics studies, however, have an increasingly broad scope and tend to contain both several types of cancer and multidimensional information for each sample and data type (platform), such as DNA copy number data, DNA methylation and miRNA transcript levels. To integrate such data, extended network modeling methods have been proposed to incorporate CNAs (2,9–11), miRNAs (12), DNA methylation (13) or clinical parameters (14). Still, the construction of comprehensive and interpretable statistical network models of *both* multiple cancers and multiple data types remains a challenging problem. The rapidly growing cancer databanks, such as TCGA, emphasize the need for refined modeling methods that work across several data modalities and diagnoses (pan-cancer analysis) (3).

### Accessible network modeling of multiple cancers

Here, we introduce a new method for large-scale integrative modeling of cancer, based on necessary extensions of a statistical method termed Sparse Inverse Covariance Selection (SICS). In its original form, SICS is a data mining method that takes a matrix of raw correlations (covariances) as input, and selects the correlations that most likely correspond to statistically direct interactions in the data. To augment this, we first introduce a novel generalization to accommodate both multidimensional cancer data and prior information, and show that the resulting optimization problem can be effectively solved for large data sets. Second, we apply the proposed method to data from several cancers obtained from The Cancer Genome Atlas (TCGA) (3). We demonstrate that networks are robustly estimated and overlap well with known pathway interactions and that the SICS model enriches for direct interactions. Third, we introduce a new tool to interpret such networks as interactive web content (cancerlandscapes.org) that enables users to explore an interactive map of multiple cancers, and that contains several functions for analyzing the structure of the network. Finally, we provide three concrete analysis examples that involve diagnosis-specific network modules, in relation to mutation data and pharmacological databases. The methodology, including models, analysis tools and software, is available through the cancerlandscapes.org site.

## MATERIALS AND METHODS

### Data sources

Data were downloaded from the TCGA http area (cancergenome.nih.gov) as TCGA level 3 data, except for mutation calls from DNA sequencing, which were downloaded as level 2 data and were standardized as described in Supplement (Pseudo-code included). URLs to all used TCGA data files are available as Supplementary Data. The TCGA data is organized into technical platforms, and we therefore chose the platform for each data type and cancer that maximized the number of patients in that dataset (Supplementary Table S2). Other sources of data integrated into downstream analyses were PathwayCommons, Gene Ontology, DrugBank, PubMed, NCBI Gene and OMIM. See Supplement for details.

### Network modeling of multiple human cancers: key principles

#### Sparse Inverse Covariance Selection

We first describe a novel integrative network modeling technique that is based on and represents a generalization of SICS. SICS is a family of statistical methods in which correlations in multivariate data are modeled as the outcome of a network of pairwise variable couplings (15). Mathematically, the network construction by SICS is formulated as the solution to a likelihood maximization problem:

\begin{equation*} \underset{\Theta }{\mbox{argmax: }} l(\Theta \mid S) - \text{penalty}(\Theta ) \end{equation*}

where *l*(Θ∣*S*) is the multivariate Gaussian log-likelihood of the network Θ, given the matrix *S* of empirical correlations between the observed variables in the data. The penalty term, usually the *L*_1_ penalty ∑_*i*, *j*_∣θ_*i*, *j*_∣, controls the size of the network (i.e. number of network links). The solution to the SICS optimization problem, Θ, is a sparse matrix corresponding to an undirected network. Each nonzero element of Θ represents a direct network connection (edge, link) between a pair of variables. Mathematically, we define a direct connection as the partial correlation between a pair of variables, i.e. the correlation that remains between the pair after accounting for all other variables. That is, the residual correlation between pairs *i* and *j* after regressing *i* and *j* on all other variables (not *i*, *j*). The benefit of SICS over correlation networks (where links correspond to correlations exceeding a threshold) is that a partial correlation structure can be described by a smaller number of links, and that such links will be more likely to reflect direct interactions. This counteracts the main flaw of correlation networks; that they contain many links that are biologically irrelevant since they are redundant (16–18). Although derived for Gaussian variables, recent works by e.g. (19) and (20) have shown that SICS can in fact provide robust and efficient estimates of sparse partial correlation for both binary and mixed variable types (Gaussian and non-Gaussian variables). Theoretical and simulation-based results in (20) motivate the following simple strategy: to include mixed variables in SICS, rank correlations are used to summarize associations for non-Gaussian variables or between Gaussian and non-Gaussian variables. Here, we therefore integrate different data types into the SICS framework by utilizing correlation and rank correlation statistics collected in large-scale correlation matrices.

#### Augmented SICS for multiple cancers and data types

**Figure 1.**
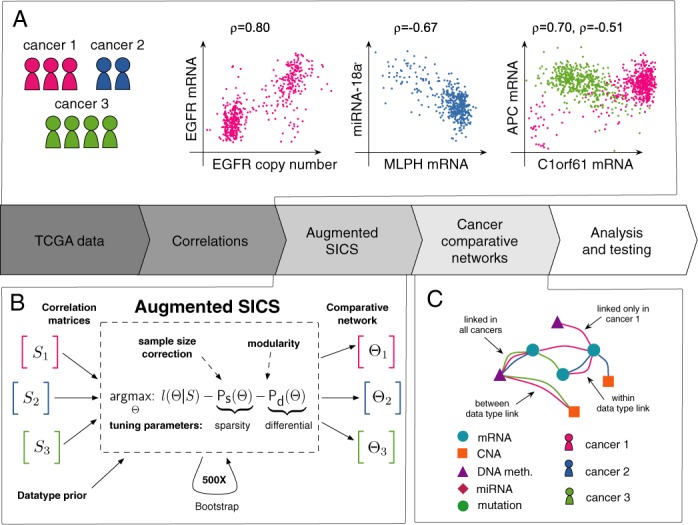
Network modeling of multidimensional cancer data: pipeline. Our modeling pipeline takes as input data from multiple -omics techniques and cancer diagnoses. (**A**) First, we compute correlations (Pearson correlation, *ρ*) between all variables in all cancers, both between the data types (e.g. CNA to mRNA) and within the data types e.g. mRNA to mRNA). We subsequently apply a novel statistical method to build a concise network that accounts for the data correlations across multiple cancers and data types. (**B**) Mathematically, this is done by solving the augmented SICS (aSICS) objective function with a fast optimization technique, ADMM (Materials and Methods). Our three-part objective function serves to (i) integrate all cancers and data types into a joint model (*l* term), (ii) produce a *sparse* network (**P**_**s**_ term) and (iii) incorporate a direct comparative element through a *differential* model penalty (**P**_**d**_ term). (**C**) The result of the optimization is a multi-cancer and multi-data type model, composed of cancer-specific networks, that depicts the statistically inferred links between the variables and relates this connectivity to the type cancer.

Whereas SICS was proposed as an improvement over correlation based analysis for biological data, including FACS (16), metabolite (17), and transcript profiles (18), its standard formulation is not suited for integrating multiple types of data across multiple cancers. Firstly, the model restricted to a single correlation matrix *S* reflecting correlations within one particular data set. Secondly, the strength of correlations between variables in heterogeneous data, such as the TCGA, is highly dependent on factors such as sample size, technical platform and the underlying biology. To address this, we propose a methodology based on the following steps (Figure 1). First, we compute raw correlations between all variables for each cancer (e.g. from TCGA datasets), resulting in a set of correlation matrices *S* = {*S*^1^, *S*^2^, …, *S*^*C*^} for each of the *C* cancer classes. The obtained raw correlations reflect both correlations within one type of data (e.g. correlations between two transcripts) as well as correlations between two types of data (e.g. correlations between a copy number alteration and a transcript, Figure 1a). In the second step, using the set of cancer-specific correlation matrices as input, we proceed to solve an extended SICS problem to obtain a corresponding set of diagnosis specific networks Θ = {Θ^1^, Θ^2^, …, Θ^*C*^} (Figure 1b). In descriptive notation, this is done by maximizing a generalized objective function:

\begin{equation*} \begin{aligned} \underset{\Theta }{\mbox{argmax: }} \underbrace{l(\Theta \vert S)}_{\mbox{likelihood}} - \underbrace{{{\bf P}}_{{{\bf s}}} (\Theta , \mbox{Prior}, \mbox{Sample size correction})}_{\mbox{network sparsity penalty}}\\ - \underbrace{{{\bf P}}_{{{\bf d}}}(\Theta , \mbox{Modularity}, \mbox{Sample size correction})}_{\mbox{network differential penalty}} \nonumber \end{aligned} \end{equation*}

where *l*(Θ|*S*) is the Gaussian log-likelihood for the networks in Θ given the correlations in *S*, **P_s_** is a penalty on network size and **P_d_** is a penalty on network differences between cancer classes (described in detail in Materials and Methods).

The proposed formulation contains a number of necessary extensions that enable integrative analysis for multiple cancers (Figure 1b). Firstly, to account for differences in the number of patients for each cancer, we modified the likelihood to include a *sample size correction*. This correction (below) is crucial for unbalanced data sets, such as the TCGA, as estimated network models are otherwise dominated by large cancer classes. Secondly, to accommodate the different types of data, we introduce a *data type dependent prior*, by which the **P**_**s**_ term is adjusted to promote particular links that are supported by external data. Our choice of prior is to reduce the penalty specifically for miRNA–mRNA links with supporting data from miRanda target prediction and links between mRNAs and the corresponding *cis*-located DNA methylation (below). Finally, to enable comparison of networks from different cancers, we apply a new modular constraint, introduced in the term **P**_**d**_.

The modular constraint stabilizes the estimated network structure across the cancers, but is also adaptive such that isolated cancer specific links can still appear in the model if strongly supported by data. Below, we present these augmentations and the exact objective function together with the parameters of the method (Table 1). To solve the proposed problem we describe an efficient gradient based algorithm that uses bootstrapping to obtain a robust network solution (Figure 1c).

**Table 1.** Description of model parameters for modeling the TCGA data

Parameter	Description	Purpose	Recommended values and comments
*λ*_1_	Sparsity parameter	Controls network size, higher values produce sparse (small) networks (Figure 2b).	User-adjustable tuning parameter in Cancer Landscapes. Applied in the range 0.7 (dense) to 0.95 (sparse).
*λ*_2_	Differential connectivity parameter	Stabilizes the network structure between cancers; an intermediate value produces higher pathway overlap (Figure 2b).	User-adjustable tuning parameter in Cancer Landscapes. *λ*_2_ in range 0.0001–0.005 gave an FDR below 10% (cf. Supplementary Figure S7b).
*δ*	Sample size correction	Balances the network size for different cancers; without correction, large classes dominate network models.	Chosen to minimize maximum size difference between any two cancer networks (Supplementary Figure S2).
*α*	Elastic net parameter	Improved performance for collinear data *α* = 1 corresponds to lasso, 0 to ridge regression.	Typical choices for *α* are 0.95 or 0.90, i.e. setting the method close to a lasso penalty). Here we use 0.95, and sensitivity analysis showed <4% effect on network composition in range 0.9–1.0 (Supplementary Figure S1).
*ω*	Structural stability parameter	Balances the network structure between cancers.	Set by the adaptive lasso algorithm (cf. Supplementary Figure S5).
*ν*	Strength of link specific prior	Reflects biological considerations, removes uninteresting links.	Recommended range for *ν* is in the between 1 (flat prior) and 0.75 (strong prior for miRNA–mRNA, CNA–mRNA and DNA methylation–mRNA links, Supplementary Figure S3, Supplementary Table S1). For sensitivity analysis in range 0.50–1.00, see (Supplementary Figure S4).

### Model construction for TCGA pan-cancer data

**Figure 2.**
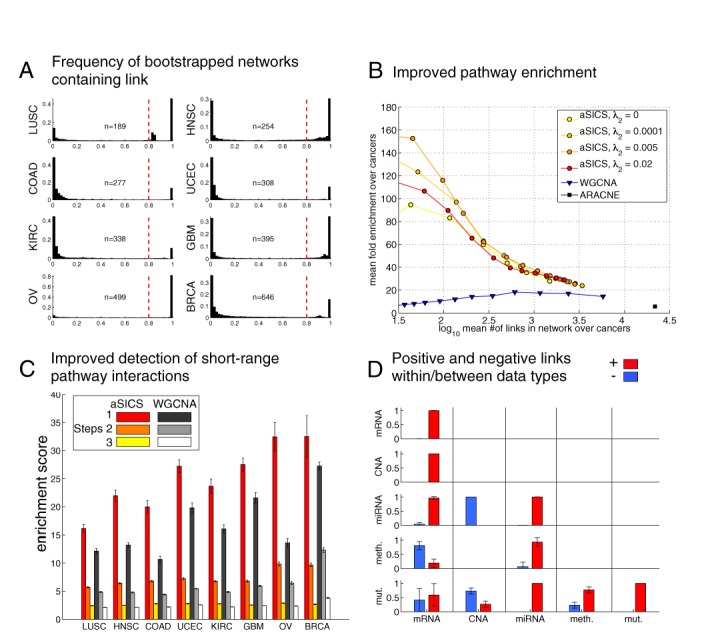
Characterization of cancer-comparative SICS. (**A**) Bootstrap analysis of network stability. The presented network models are created as a summary of 500 networks made from pseudo-bootstrapped TCGA patient data. The histograms display the frequency of the presence of specific links over the bootstrapped networks for each cancer. The final network is made by selection of links present in >80% of the bootstrap networks (red threshold line). (**B**) Characterisation of Cancer comparative SICS by pathway overlap, measured as fold enrichment of known links from the database PathwayCommons (Materials and Methods). For a range of network sizes (50–3400 links), augmented SICS achieved 30-fold to 160-fold enrichment. The enrichment of known links depended on both network size (x-axis) and on the cancer differential penalty (**P**_**d**_, tuned by *λ*_2_, see Materials and Methods). (Figure 2b, signed rank test *P* < 0.01). As a point of reference, the corresponding enrichment was not as high for a standard correlation network (WGCNA, signed rank test, *P* ≤ 0.0078). A nonparametric reference method (ARACNE) produced considerably denser networks, also at the highest stringency setting, making the comparison difficult. (**C**) Analysis of direct vs indirect links. We stratified the analysis of pathway overlaps (B, above) into short-range interactions (direct links in PathwayCommons) and long range interactions (second and third order indirect links in PathwayCommons). This showed a marked difference in performance between SICS and WGCNA for direct links (*P* < 0.0001 for all eight cancers), and a less pronounced but still significant, when comparing indirect (two or three steps) interactions (Figure 2c, *P* < 0.0001 except for breast cancer). Error bars = 99% CI. (**D**) Link signs of the SICS model are broadly consistent with biological mechanism. Bar charts display the mean proportion over cancers of negative (blue) and positive (red) links within and between different data types. Eighty percent of methylation-DNA links are negative (plausibly reflecting *cis*-acting methylation-mediated suppression of transcription) and CNA-mRNA links are positive, plausibly reflecting elevated transcriptional rates of genes with multiple chromosomal copies.

The model, and estimation procedure are introduced in the results section. From the TCGA data we computed correlation matrices for the joint set of mRNA, miRNA, CNA, DNA methylation and point mutation variables in each cancer. For different values of sparse penalty (*λ*_1_) and differential connectivity penalty (*λ*_2_), we run 500 bootstrap simulations, each time solving the augmented SICS global objective function (below). Pseudo-code for the construction of a correlation matrix from bootstrapped data is available in the Supplement. We construct bootstrap summary statistics as follows: for each link, we compute (i) the detection frequency (proportion of bootstraps where it is included in the network) and (ii) for each pair of cancers, the frequency of differential connectivity (proportion of bootstraps where the link attains a different value for the two cancers). Examples of histograms of bootstrap frequencies for all links are shown in Figure 2a and Supplementary Figure S7. A robust, final network estimate is produced by thresholding the bootstrap frequencies, retaining links that appear frequently across bootstraps and defining such links as differential between pairs of cancers if they exhibit a high bootstrap frequency of being differential. The networks analyzed here and presented through cancerlandscapes.org were obtained using frequency thresholds 80% (for retaining links) and 60% (for differential links), respectively. In the Supplement we also show that the false discovery rate (FDR) for detection of differential links is highly robust with respect to the choice of threshold. The model construction was done using parameter values set according to Table 1. Key parameters controlling sparsity and differential connectivity were varied across a wide range of values: *λ*_1_ between 0.7 and 0.95 (sparse penalty) and *λ*_2_ between 0 and 0.02 (differential connectivity penalty); we thus obtained one network for each combination of *λ*_1_ and *λ*_2_. When choosing values for these parameters, the user should consider that higher values of *λ*_1_ produce networks that are more enriched for known interactions (cf. Figure 2) but that also are smaller. The user should therefore consider the tradeoff between coverage of many genes and mutations and accuracy/interpretability of the network. For the parameter *λ*_2_ the validation against known pathway links suggests that a small, non-zero value produces better results. Setting the *λ*_2_ parameter to a very high value leads to a network that almost exclusively contains common links between the cancers. In the Supplement we show that FDR for detecting differential links, including cancer specific connections, is minimized for *λ*_1_ in the range 0.7–0.8 and *λ*_2_ in the range 0.0025–0.005. These and other tuning parameters are discussed below, in Table 1, and further analyzed in the supplement.

### Modeling: optimization problem and parameter settings

#### Network modeling by augmented SICS

The network estimation takes a set of correlation matrices *S*^*c*^, for cancer diagnoses *c* = 1, 2, …, 8, each based on *n*_*c*_ patient samples. Given this input, we maximize the penalized likelihood function

(1) \begin{equation*} \begin{aligned} \max _{\Theta ^c, c=1,\cdots ,C} \mbox{ } \sum _{c=1}^C \underbrace{ n_c(\log \det \Theta ^c - \mbox{tr} (S^c\Theta ^c))}_{negative \ log-likelihood}\\ - \underbrace{\sum _{c=1}^C\sum _{i \ne j}\lambda _1^c \nu _{ij } (\alpha \mid \theta _{ij}^c \mid +(1-\alpha ) (\theta _{ij}^c)^2)}_{sparsity \ constraint}\\ - \underbrace{ \sum _{c<c^{\prime }}\sum _{i \ne j} \lambda _2^{cc^{\prime }} \omega _{ij}^{cc^{\prime }} \mid \theta _{ij}^c-\theta _{ij}^{c^{\prime }} \mid }_{differential \ connectivity \ constraint}, \end{aligned} \end{equation*}

in which the inverse covariance matrices Θ^*c*^ denote cancer specific networks for each cancer class *c*. Matrix element $\theta _{ij}^c$ represents the link strength between nodes (variables) *i* and *j* in cancer class *c*, with $\theta _{ij}^c=0$ if and only if nodes *i* and *j* are conditionally independent given all other nodes. Important augmentations in our methodology compared to standard SICS and multi-sample generalizations (16,21) are:

- Correction for the different sample sizes is defined by $\lambda _1^c=\lambda _1 n_c^e$ and $\lambda _2^{cc^{\prime }}=\lambda _2 \frac{2 n_c^e n_{c^{\prime }}^e}{n_c^e+n_{c^{\prime }}^e}$, where $n_c^e=\bar{n}^\delta n_c^{(1-\delta )}$ and $\bar{n}=\frac{1}{C}\sum _{c=1}^c n_c$. *n*_*c*_ is the mean sample size over data types for cancer *c*. The tuning factor *δ* controls the degree of sample size correction and is chosen to produce similar sparsity levels across all cancer classes, which by simulation was found to also maximize the TPR (true positive rate) for different fixed levels of FPR (false positive rate) (Supplement). If sample size correction is not utilized, large cancer classes dominate the network.
- Prior to facilitate detection of miRNA targets, *cis* methylation effects and impact of mutations.
  The global objective function includes a *link specific prior*, *ν_ij_*, which is designed to tune the sparsity penalty for forming a link between network nodes *i* and *j*. The sparsity penalty for link element (*i*, *j*) is defined as *λ*_1,*ij*_ = *λ*_1_ × *ν_ij_*, where *λ*_1_ is a common factor that controls the overall sparsity of the network, and *ν_ij_* take on three possible values: 1, *u* (<1), or ∞. The motivation for this choice of prior is that it can serve to emphasize features of the model that are either more likely, based on prior information, or are of higher biological interest to the end user. In such cases *ν_ij_* is set to the value *u* < 1. This reduced penalty is applied in the following situations:
  - between miRNAs with their predicted mRNA targets, as defined by miRanda (22) prediction (MicroCosm Targets Version 5 (23), http://www.ebi.ac.uk/enright-srv/microcosm/htdocs/targets/v5/). A strong rationale for using such a prior is the observation that miRanda and other target predictions are enriched among miRNA–mRNA correlations with strong negative values (24). We further analyze the information content of the prior in Supplementary Table S1 and its effect on network structure in Supplementary Figure S3 and S4.
  - between *cis* localized methylations probes with their corresponding mRNA, as defined by associations between genes and methylation probes provided in the level 3 data by TCGA. This choice is motivated by the belief that such *cis*-localized probes are likely to be involved in transcriptional suppression. Many of the detected links between promoter methylations and mRNAs do indeed have a negative sign, consistent with this expectation (Figure 2).
  - between all interactions involving a point mutation. This choice is motivated by the assumption that point mutations are key determinants of downstream epigenetic and transcriptional events; and, (iv) to model the assumption that the effect of CNAs on transcription is only via cis-effects, i.e. mRNAs can only be linked to CNAs at their coding locus, which is done by setting νij = ∞ for all trans-interactions that involved CNA and an mRNA, and νij = 1 for all cis interactions. We chose the value u = 0.75 in our analyses, and found upon inspection that this prior ensures a balanced model, with involvement of the different data types. Using no prior at all produced results with an extensive number of links between CNAs in close genetic proximity and methylation probes in close genetic proximity, which we regard as a less informative network. We therefore also set νij = ∞ for such connections. We performed a simulation study to investigate the impact of this last restriction and found that while the network weights for other network connections were altered, the network structure itself was not much affected. While a prior formally does not require validation (because it reflects a belief), changing the prior structure will likely be useful to bring forward different aspects of the data.

In addition, the prior is used to model the assumption that the effect of CNAs on transcription is only via *cis*-effects, i.e. mRNAs can only be linked to CNAs at their coding locus, which is done by setting *ν_ij_* = ∞ for all *trans*-interactions that involved CNA and an mRNA, and *ν_ij_* = 1 for all *cis* interactions. We chose the value *u* = 0.75 in our analyses, and found upon inspection that this prior ensures a balanced model, with involvement of the different data types. Using no prior at all produced results with an extensive number of links between CNAs in close genetic proximity and methylation probes in close genetic proximity, which we regard as a less informative network. We therefore also set *ν_ij_* = ∞ for such connections. We performed a simulation study to investigate the impact of this last restriction and found that while the network weights for other network connections were altered, the network structure itself was not much affected. While a prior formally does not require validation (because it reflects a belief), changing the prior structure will likely be useful to bring forward different aspects of the data.

- Modularity constraints on the similarities across cancers are designed to generate more biologically plausible networks. This is achieved by encouraging neighboring links to be equal for cancer pairs *c* and *c*′ through the term $\omega _{ij}^{cc^{\prime }}$, thus limiting isolated, spurious differential links. The adaptive factor $\omega _{ij}^{cc^{\prime }}$ is designed to improve the stability of the network estimates and generate interpretable networks. This is done by a two-step adaptive lasso ((25)) method, in which preliminary network estimates (obtained using $\omega _{ij}^{cc^{\prime }}=1$) are used to update $\omega _{ij}^{cc^{\prime }}$ to a new value obtained from the initial network estimate $\tilde{\Theta }$. The purpose of the update is to encourage all links within a module, or local sub-network, to exhibit the same link commonality or link differential connectivity properties across cancers. Since the penalty is adaptive, strong differential signals in the data sets will still produce differential connectivity and the modularity is only encouraged when it is supported by data.

#### Optimization and method parameters

We solve the above optimization problem, by a new algorithm based on nested ADMM (Alternating Directions Method of Multipliers (26)). ADMM is a robust gradient-based method suitable for constrained convex optimization (here, log-likelihood and two penalty functions) and converges to a global optimum under weak conditions. Source code in Matlab is available as Supplementary files. To produce stable and robust network models, we resample patients and re-estimate the networks. This is repeated 500 times and the network estimates are aggregated as follows; (a) links that appear with high frequency (at least 80% of models) across bootstraps are retained, and, similarly, (b) frequency statistics on links differing or coinciding across subsets of cancers are used to form the final comparative network (Supplement). An investigation of the stability of networks based on different number of bootstraps, which showed that stability does not increase notably after around 200 bootstraps, indicated that 500 bootstraps is more than sufficient. (Supplementary Figure S6).

The optimization is governed by a set of tuning parameters, each with a distinct purpose/function (Table 1). The two key parameters are *λ*_1_ (sparsity parameter) and *λ*_2_ (differential connectivity parameter). These two parameters control the network size and emphasis on shared mechanisms between cancers, respectively. *λ*_1_ and *λ*_2_ are not set to a single optimal value, but the model is instead constructed for a broad range of such values, which are available in Cancer Landscapes. Overall, a higher *λ*_1_ gives a smaller network, which is more enriched for true pathway links, as shown in Figure 2b, cf. (2). The analysis of network modules in the main paper was performed using *λ*_1_ = 0.7 and *λ*_2_ = 0.005, except Figure 2a, in which we use λ_2_ = 0 to keep the models independent (the motivation for this setting is that an optimum was reached in terms of PathwayCommons overlap, Figure 2b). In addition, estimated FDR for differential connectivity was shown to be controlled well <5% for these parameter values (Supplement). Figure 2a clearly illustrates the stability of the network estimation. The ”U-shape” frequency histograms show that links are persistently present or absent across bootstraps. Similar results are also observed for frequency of differential connectivity (Supplementary Figure S7). Small changes in parameter values did not substantially change the networks (i.e. cluster structures are largely preserved). Results for other settings are available through the web system. In addition to *λ*_1_ and *λ*_2_ , an important parameter is the sample size correction *δ*, which is set by an empirical method that aims to maximize the global true positive rate by choosing a *δ* for which the networks of different cancers have the most similar size (Supplementary Figure S2). The parameter *α* is the standard elastic net parameter, set to 0.95 (Supplement). The parameter *ω* is set by an empirical method (Supplement) and tests on TCGA data support that *ω* > 0 improves stability and pathway overlap (Supplementary Figure S5). Some of the values are data set specific, and will require some adaptation for other * = (R package glmnet vignette http://web.stanford.edu/∼hastie/Papers/Glmnet_Vignette.pdf, and (27,28)).

#### Pathway enrichment scoring

For analyses in Figure 2b, estimated networks were compared against pathway databases HPRD (hprd.org), NCI (pid.nci.nih.gov), REACTOME (reactome.org) and IntAct (www.ebi.ac.uk/intact) downloaded from Pathwaycommons.org. We mapped gene identifiers in the databases to our set of variables. We then computed the length of the shortest path *d*_*ij*_ between nodes *i* and *j* using Johnson’s algorithm (29). We define the pathway enrichment of a network Θ as the ratio between the observed overlap and the expected overlap for a permuted network, calculated over 100 simulations (Supplement). In our comparison to correlation networks, we compare the enrichment of direct (step length 1) and indirect (step length 2, 3) PathwayCommons links for augmented SICS (sparsity parameter *λ*_1_ = 0.7, differential connectivity parameter *λ*_2_ = 0.005, mRNA–mRNA links only) and WGCNA networks of similar size for each cancer.

### Cancer Landscapes tool

The Cancer Landscapes web application (cancerlandscapes.org) uses HTML5 technologies and Javascript to give a rich user experience and is compatible with all modern web browsers (Chrome, Firefox, Safari, Opera, IE 9+). Some of the benefits of these technologies include: high performance visualization using the HTML5 canvas element, asynchronous server requests to load data seamlessly in the background and cross-platform compatibility. Although the system works well in all modern web browsers, we recommend the use of Google Chrome, since it shows better performance across core technologies. The network drawing in the application is built on the free **sigma.js** package (http://www.sigmajs.org) but modified to suit the particular needs of visualizing multi-cancer networks. Other software packages used include **jQuery** (http://www.jquery.com) for extended JavaScript functionality and **d3js** (http://www.d3js.org) for comprehensive plotting capabilities.

### Analysis of network modules and survival associations

We applied hierarchical clustering of the network, using topological overlap as the distance measure between nodes and choosing number of clusters by silhouette width analysis (Supplement). Pathway annotations were computed by Fisher’s exact test. *P*-values were adjusted by Benjamini-Hochberg correction and considered significant if <0.05. For enrichment of survival associated nodes the same test was used. To label nodes as survival associated in the enrichment analyses, we used a Kaplan–Meier log-rank *P*-value cutoff of 0.05 (this is a deliberately inclusive threshold to avoid very low counts in enrichment testing).

## RESULTS

### Sparse Inverse Covariance Selection for multiple cancers and datatypes

We first developed a novel integrative network modeling technique that is based on and represents a generalization of SICS. The methodology is described in detail in Materials and Methods section, whereas this section emphasises the key principles. Before network construction, we compute correlations for all variables in the dataset, both within each data type (e.g. mRNA–mRNA correlations) and between data types (e.g. CNA–mRNA correlations) (Figure 1a). The full set of correlations for each cancer (cancers are given index 1, 2, ..., *k*) are subsequently organised into a correlation matrix *S*_1_, *S*_2_, ..., *S*_*k*_. The computational task that we seek to solve is to identify which correlations in these matrices correspond to direct variable dependencies. We do this by a new generalisation of the SICS methodology; in essence, given the correlation data, we solve a statistical optimisation problem to obtain a set of diagnosis specific networks Θ_1_, Θ_2_, ..., Θ_*k*_ (Figure 1b). In these networks, nodes represent different types of variables (e.g. particular mRNAs, CNAs and miRNAs) and connections represent identified links in different cancers (Figure 1c). The benefit of SICS over correlation networks (where links correspond to correlations exceeding a threshold) is that a partial correlation structure can be described by a smaller number of links, and that such links will be more likely to reflect direct interactions. This counteracts the main flaw of correlation networks; that they contain many links that are biologically irrelevant since they are redundant (16–18). Furthermore, unlike standard correlation networks, estimation is done jointly (for all the cancers and data types simultaneously) to produce a more stable result (see Materials and Methods). For the method to be applicable to cancer data across several diagnoses and data types, we have introduced a number of necessary generalisations. These include a new modification of the SICS equations to achieve a sample size correction, which ensures that the estimated network models are not dominated by large cancer classes (Materials and Methods and Supplement). Secondly, to accommodate the different types of data in a biologically meaningful way, we introduce a *data type dependent prior*, to promote particular links that are supported by external data. Our choice of prior is to reduce the penalty specifically for miRNA–mRNA links with supporting data from miRanda target prediction and links between mRNAs and the corresponding *cis*-located DNA methylation. In support of this particular prior, we note that miRanda predictions are enriched among miRNA–mRNA pairs with negative correlations (Supplementary Table S1), and that constructing the network with a flat prior tends to enrich for prior links by up to 100-fold (Supplementary Figure S4). In Methods, we present the details of these augmentations, as well as the exact objective function that is solved in the SICS problem, together with the parameters of the method (Table 1). To solve the proposed problem we describe an efficient gradient based algorithm that uses bootstrapping to obtain a robust network solution (Figure 1c). The proposed method is a generalised framework for integrative modeling; depending on signals in the data, the method will detect links as present in multiple cancers, a subset of cancers, or a single cancer (Figure 1c). In the supplement, we show that the method’s performance in assigning each link to a distinct pattern of cancers (e.g. ‘connected in all cancers’, or ‘breast cancer specific’) can be estimated as a FDR from the bootstrap simulations. As illustrated below, it serves as a tool to study both particular and general aspects of cancer.

### Application to TCGA data enriches for direct interactions

To characterize this framework, we applied it to TCGA for eight cancers: glioblastoma multiforme (GBM), breast cancer (BRCA), ovarian carcinoma (OV), lung squamous cell carcinoma (LUSC), colon adenocarcinoma (COAD), uterine carcinoma (UCEC), kidney clear cell carcinoma (KIRC) and head and neck squamous cell carcinoma (HNSC). This set of diagnoses represents the cancers for which data for at least 200 patients was available at the time of data download. Together, the selected diagnoses cover many anatomical locations and represent a substantial fraction of both cancer incidence and mortality in humans (http://cancergenome.nih.gov/cancersselected). We first solved the generalized SICS problem for the eight cancer data set, using 500 bootstrap runs and a biologically informative link-specific prior (Supplementary Figures S4 and S6). In all eight cancers, we detected network links that were robustly present in a high proportion (at least 80%) of the 500 bootstrap runs (Figure 2a). Network links were declared differential between subsets of cancers if differential values were observed in a high proportion (at least 60%) of the bootstraps (Supplementary Figure S7). Retaining such links resulted in an eight-cancer network that connected 110 point mutations, over 600 connected DNA copy number aberrant gene loci, over 3200 mRNAs, 200 miRNAs and over 1600 methylation sites. Using network quality measures described in (2), we detected a good overlap with known links from PathwayCommons (Figure 2b and c) and robust estimation properties (Supplement). Specifically, the generalized SICS procedure achieved 30-fold to 160-fold enrichment of known PathwayCommons links (Figure 2b). As a point of reference, we calculated the same level of pathway overlap for a TCGA-derived correlation network (here calculated by the WGCNA method). The correlation network showed a lower level of pathway overlap of 10–20-fold (Figure 2b, *P* = 0.008, signed rank test). A key statistical distinction between the SICS-derived and correlation-based networks is that the former should be more prone to enrich for direct interactions. For the TCGA data, this effect could be observed by stratifying the pathway overlaps into short-range interactions (direct links in PathwayCommons) and long range interactions (2nd and 3rd order indirect links in PathwayCommons). This showed a marked difference in performance between SICS and WGCNA for direct links (*P* < 0.0001 for all eight cancers), and a less pronounced but still significant, when comparing indirect (two or three steps) interactions (Figure 2c). Analysis of network robustness indicated that the estimation of SICS networks is as stable as estimation of networks using correlation networks and other approaches (Supplement).

In summary, the proposed method enables the integration of several TCGA datasets into a statistically robust multi-cancer SICS-based network. The method addresses some of the shortcomings of existing correlation-based and naive SICS-based methods and performs well in tests on TCGA data in terms of robustness and pathway overlap. It is important to be mindful of the fact that all statistically derived networks are fundamentally a *statistical summary* of the data. Thus, while we can empirically demonstrate a tendency to overlap with known pathway links (e.g. Figure 2b), the detected links are not necessarily evidence of mechanistic or physical interaction. It is therefore crucial that the user of Cancer Landscapes interprets links in a biological context. For instance, 80% of methylation-DNA links are negative (plausibly reflecting *cis*-acting methylation-mediated suppression of transcription) and CNA–mRNA links are positive, plausibly reflecting elevated transcriptional rates of genes with multiple chromosomal copies (Figure 2d). As is detailed in the Materials and Methods and Supplement, a key feature of the method is that different settings of the optimization parameters will bring forward different aspects of the data, and an important area for future study will be to further develop the network prior. Another aspect to consider in the interpretation of the models is that technical variation in the data can affect the results. For instance, the current version of TCGA exhibits technical heterogeneity in the sense that different methods were used to collect mRNA profiling data and DNA copy number data, and future standardization of TCGA data will likely improve results further. The source code in Matlab is available as Supplementary files, and we foresee that our method could have interesting applications beyond cancer studies, e.g. integration of multiple GWAS studies or modeling of multiple metagenomic datasets.

### Interpretation of multi-cancer network models using Cancer Landscapes

Next, we describe a new visualization technique, in which the multi-cancer network is made available as interactive web content for analysis through an interface available at cancerlandscapes.org. Combining features of a data access portal and a network analysis tool, this resource is designed to (i) enable easy access to cancer comparative models, (ii) provide a clear and intuitive visualization of the networks and (iii) enable analysis of the network in terms of pathway information, clinical data in TCGA and the underlying molecular measurements. Thus, the tool has a different spectrum of functions compared to existing tools for TCGA data access such as the cBio portal (30,31), Cancer Genome Browser (32)) or user-installed programs for data analysis such as Cytoscape (33,34) and Integrative Genomics Viewer (35) (comparison table in Supplement). In the next sections, we describe how the multi-cancer models can be accessed through cancerlandscapes.org, and exemplify its use for cancer research, with examples relevant for functional interpretation, discovery of subtypes defined by joint mutational events and identification of candidate of drug targets.

#### Accessing the system

**Figure 3.**
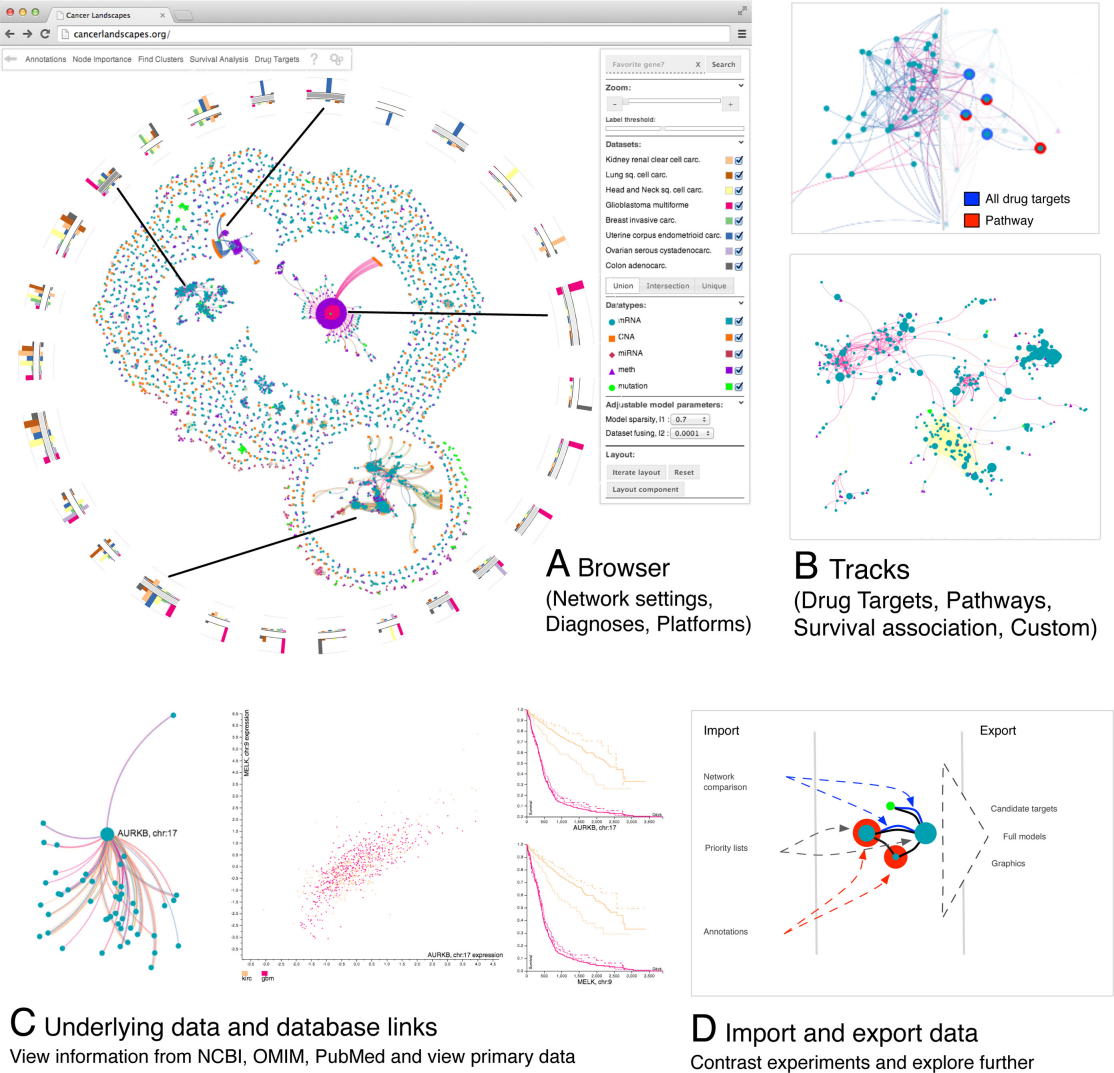
The Cancer Landscapes system is available at cancerlandscapes.org and enables users to access and explore pan-cancer network models built from TCGA data and other sources. The system provides a wide range of functions tailor made for analyzing these models, allowing the user to: (**A**) View a cluster summary of the network showing regions that contain an overrepresentation of survival biomarkers or pathway annotations. (**B**) Highlight nodes based on available annotations (PathwayCommons (54), KEGG (55)), GOslim (56) and DrugBank (52,53)) or user supplied lists. (**C**) Rank nodes according to network centrality measures, survival *P*-values or user supplied lists, to view the distribution of these rankings across the network. (**D**) Import node lists or small networks for comparison with the provided models, and exporting the full models or target lists for further analysis in other software.

A user (cancerlandscapes.org) starts by selecting one of the multi-cancer models for further analysis. The system subsequently loads the model and starts the network browser (Figure 3a), in which the different data types and cancers are encoded as specific shapes and colors (cf. Figure 1). In this exploration view, the user can bring forward parts of the networks by toggling the different data types, adjusting the optimization parameters, organizing the network, and zooming in to access primary data (Figure 3a). Next, we give three examples of how the system can be used to explore network modules, new co-occurring mutations, and drug targets, respectively.

#### Example 1: network modules with general versus cancer specific connectivity

**Figure 4.**
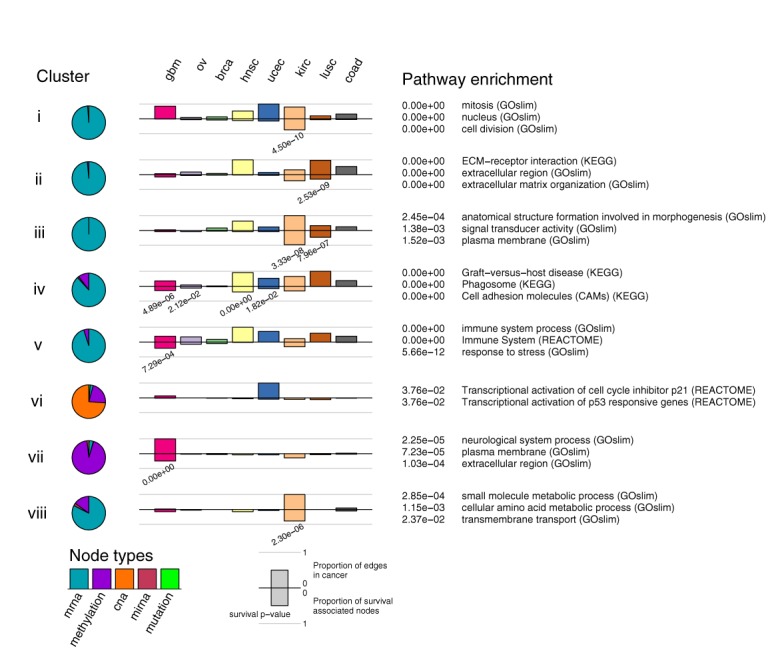
Functional annotation of network modules. Cancer Landscapes identifies and characterizes network modules across multiple cancers. Here, we display a selection of 8 clusters, organized in rows, displaying a number of properties. The pie charts (left) display the distribution of the data types of the nodes included in the cluster. The middle panel displays two properties: the bars above the middle line represent the proportion of links present in the cluster for the different cancers, and the bars below the middle line represent the proportion of significantly associated survival nodes (Materials and Methods) in each cancer. The right panel lists significantly (*P–* < 0.05, BH corrected) associated pathways from PathwayCommons and GOslim.

From the analysis menu, two categories of analysis are available: modules and tracks (Figure 3). The former is a set of functions to analyze and visualize structures in the network. This is done by clustering of the network into modules (Materials and Methods), which are subsequently visualized as concise bar charts that summarize (i) the cancer diagnoses connected in that module, (ii) the functions of the involved genes, and; (iii) to what degree the module is enriched for survival associated (Kaplan–Meier log-rank test, Materials and Methods) nodes (Figure 4).

#### Multiple cancer network modules

Exploring such patterns in the TCGA-derived network, the system detected two broad classes of modules. One set of modules comprise nodes that were connected in multiple cancers and tend to contain genes involved in characteristic (hallmark) processes of human cancer (36,37). Examples (roman numerals indicate charts in Figure 4) include mitosis (i), tumor vasculature (ii and iii), and immune responses (iv and v). Although they were connected in multiple cancers, the modules differed in terms of their survival association. For instance, an association between cell cycle genes and survival was found in kidney cancer (i), whereas immune response was associated to survival in glioblastoma, head and neck, ovarian and uterine cancers (iv, v). Angiogenesis (ii, iii), in turn, was associated with survival in kidney and lung cancer. These differences may reflect lineage or tissue dependent differences in growth dynamics and disease etiology, c.f. (38,39).

#### Cancer specific network modules

In addition to modules that were connected in several cancers, the multi-cancer network contained modules that were predominantly or exclusively connected in a single cancer. Examples of such cancer-selective modules were found in glioblastoma (Figure 4, vii), uterine cancer (vi) and kidney cancer (viii). Interestingly, the cancer selective modules often contained survival associated nodes in the most frequently connected cancer. Examples include a glioblastoma (GBM)-selective module in which *IDH1* mutation was directly linked to over 600 *cis*-located promoter methylations (Figure 4, vii). This possibly reflects the CpG island hypermethylator subclass of glioblastomas (40), which is positively correlated with survival and for which *IDH1* is an important regulator (41). Additional cancer specific network modules (Figure 4 and Supplement) reflect TP53 point mutation linked to a number of TP53 targets in uterine cancer (Figure 4, vi), and enrichment of solute carrier encoding genes in kidney cancer (Figure 4, viii).

Thus, the module summaries generated by the system helps the user get an overview of the complex network model, and should serve as a starting point to explore parts of the network with broad or selective representation, respectively.

#### Example 2: co-occurrence of *IDH1* mutations and 11p15.3-5 deletions in glioma

To illustrate how a module can be analyzed in greater detail, we next focus on the the glioblastoma-specific module defined by *IDH1* mutation (Figure 4, module vii). The model detected a link between the presence of a mutation in *IDH1* and a CNA in 14 genes on chromosome 11: *AP2A2*, *APBB1*, *CD81*, *FXC1*/*TIMM10B*, *HBE1*, *LSP1*, *MRPL23*, *PNPLA2*, *POLR2L*, *RHOG*, *TOLLIP*, *TRIM3*, *TRIM5* and *TRIM68*. These genes map within 5.7 Mb of each other at the end of the short arm of chromosome 11, within cytobands 11p15.3–11p15.5. Deletions of chromosome 11p loci are frequent in different cancers, and loss of heterozygosity (LOH) in a 7 Mb region spanning cytobands 11p15.4-5 was previously associated with malignant glioma (42,43). LOH within a common minimal 130 kb interval in this region identified the tripartite motif protein 3 (*TRIM3*) as a candidate tumor suppressor gene involved in glioma progression (44). While co-occurrence of 19q loss and *IDH1* mutations has been found to diminish the survival advantage conferred by the *IDH1* mutation in low-grade glioma (45), nothing has been reported so far about *IDH1* and 11p15 loss co-occurrence.

**Figure 5.**
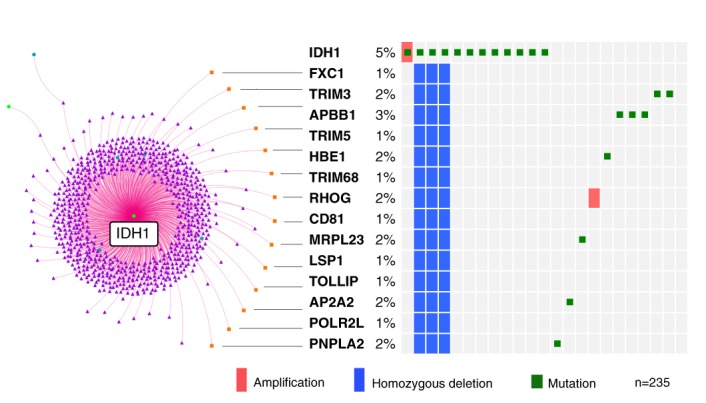
11p15 deletion co-occurs with *IDH1* mutation. Oncoprint plot (31) illustrating co-occurrence of *IDH1* mutations and homozygous deletions in 14 genes located in the 11p15 region found to be correlated by our model. The percentages indicate the proportion of samples with an alteration. The samples with any alteration correspond to one row in the table. All high-grade glioma patients carrying homozygous deletions in these genes have *IDH1* mutations. While the co-occurrence is present in the majority of low-grade glioma patients with both types of mutations, it is not observed in the other types of cancers (Supplementary Figure S9).

To assess this finding in more detail we investigated the co-occurrence of *IDH1* mutations and LOH in the 14 genes selected by the model in several cancers using the oncoprint representation made available by the cBio Portal (31). Homozygous deletions in these genes co-occur with *IDH1* mutations in glioblastoma patients (Figure 5, Fisher test *P*-value < 0.0001 for the 14 different CNAs, co-occurrence odds ratio > 10), and to a lesser extent in lower grade glioma (co-occurrence odds ratios 2–10 for 10 CNAs, but not significant), but not in uterine, breast, ovarian, head and neck, lung, colon or kidney cancer patients (Supplementary Figure S9). In spite of the limited number of cases with combined *IDH1* mutation and 11p deletion, it would be interesting to explore this finding in larger cohorts given the importance of *IDH1* mutations in some of the emerging subtype classification systems developed for glioma (40,46–49).

In addition to the example discussed, the model contains additional linked CNA regions, making it possible to associate chromosomal regions to general cellular processes in particular cancers. For instance, we found a high number of associations between the mitosis-associated network module (Figure 4i), CNAs located on chromosome 8 (*FNTA*, *GOLGA7*, *WHSC1L1*, *DDHD2*, *BAG4*, *LSM1*, *ASH2L*, *BRF2*, *PROSC*, *PPP2CB*, *PPP2R2A*, *CHMP7*, *XPO7*, *CNOT7*), previously found in breast cancer (50), and an amplicon on chromosome 3 (*DVL3*, *SENP2* and *ABCF3*), previously found in lung cancer (51).

#### Example 3: overlaying survival, drug target and other information onto the multi-cancer network

**Figure 6.**
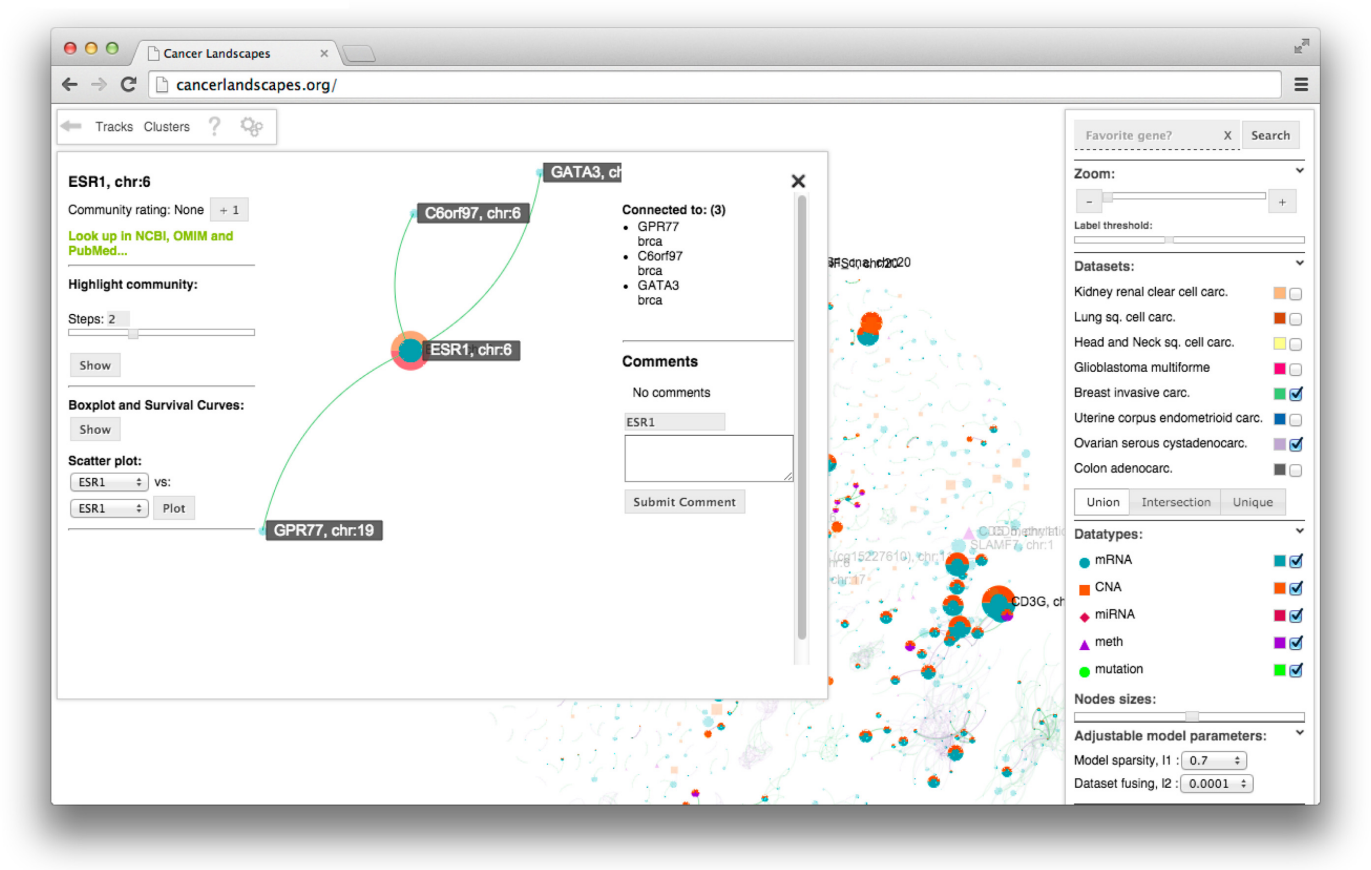
Using Tracks to highlight survival associated drug targets. The Cancer Landscapes system was used to highlight known drug targets (from DrugBank) and survival associations (from TCGA). In the resulting graph, the size of each node is made proportional to the negative logarithm of the Kaplan–Meier *P*-value estimated from the TCGA cohort. In this example, the estrogen receptor (*ESR1*) is connected to two recently described modulators of estrogen receptor signaling (*EYA2* and *GATA3*) (57,58), but also the orphan G-protein coupled receptor *GPR77*.

In addition to the module-oriented analysis, Cancer Landscapes uses a group of functions termed *Tracks* to superimpose relevant gene-specific information onto the network. Tracks can be externally uploaded (e.g. lists of differentially expressed genes or lists of siRNA screening hits). Several sources of information are also available by default, including the drug target database DrugBank (52,53), and pathway and annotation databases such as PathwayCommons (54), KEGG (55) and Gene Ontology (56). To illustrate this, we used the Cancer Landscapes tool to simultaneously mark both the known drug targets in the network (marked by colors) and strength of survival associations (marked by node sizes, proportional to the negative logarithm of the Kaplan-Meier *p*-value). Applying this view to the ovarian and breast cancer portions of the network brought forward several marked nodes, one of which is the estrogen receptor *ESR1* (Figure 6). In this particular example, *ESR1* was linked to both known modulators of estrogen receptor signaling (*GATA3*, *EYA2*) (57,58), as well as a third gene, *GPR77*, that is not previously implicated in estrogen receptor function and could be explored by directed studies. In addition to using the system to explore drug targets, the tracks function can be used to score genes or to map externally defined gene sets onto the networks (e.g. hits from siRNA screens, or lists of differentially expressed genes).

## DISCUSSION

As public repositories of cancer -omics data continue to grow, accurate and accessible integrative analysis will be one of the key challenges in cancer research. The strategy proposed here combines principled data-driven modeling with user-friendly public access of results, and is a novel way of making TCGA and similar data available to the community. As we have pointed out, data integrative models are best seen as useful summaries of data that aim to provide a global perspective on the biological mechanisms involved, and enables formulation of mechanistic hypotheses, but should not be assumed as direct mechanistic models of the underlying cell biology.

As was illustrated by the examples of TCGA-derived network modules, the modeling strategy presented detects both common links that appear in multiple cancers, and links that appear in a subset of cancers, or a single cancer. Interestingly, at a controlled FDR of <10% (Supplement), the TCGA data seems to favor a model in which links have either a very broad representation across cancers, or are present in only a single cancer (Figure 4). This can be due to the fact that the spectrum of cancers analyzed still is relatively small (eight diagnoses), and application to data from more diagnoses will likely detect modules in subsets of the cancers. To further understand the impact of diverse mutations across cancer types (59) it would thus be interesting to generalize the proposed model to represent both different cancer diagnoses and molecular subtypes within each cancer, reserved for future work.

In an ongoing effort, the size and scope of cancerlandscapes.org is being expanded, as TCGA and other data sources grow. An important future extension will be to enable users to model their own data in relation to TCGA data and other data sources. We anticipate that Cancer Landscapes will become a useful data mining portal for cancer research that combines statistically rigorous network modeling with user-friendly model accessibility and interpretation.

## SUPPLEMENTARY DATA

Supplementary Data are available at NAR Online.

SUPPLEMENTARY DATA
