# Supplementary material for: Efficient exploration of pan-cancer networks by generalized covariance selection and interactive web content
Source: Nucleic Acids Res. 2015 May 7;43(15):e98. doi: 10.1093/nar/gkv413 (PMC4551906; doi:10.1093/nar/gkv413)
Supplement: SUPPLEMENTARY DATA [file supp_43_15_e98__index.html]

Efficient exploration of pan-cancer networks by generalized covariance selection and interactive web content — Efficient exploration of pan-cancer networks by generalized covariance selection and interactive web content — SUPPLEMENTARY DATA 

# Efficient exploration of pan-cancer networks by generalized covariance selection and interactive web content

## SUPPLEMENTARY DATA

- SUPPLEMENTARY DATA
- SUPPLEMENTARY DATA
- SUPPLEMENTARY DATA
