# Supplementary material for: Efficient exploration of pan-cancer networks by generalized covariance selection and interactive web content
Source: Nucleic Acids Res. 2015 May 7;43(15):e98. doi: 10.1093/nar/gkv413 (PMC4551906; doi:10.1093/nar/gkv413)
Supplement: SUPPLEMENTARY DATA [file supp_gkv413_nar-03235-met-n-2014-File008.pdf]

1 Supplementary Methods

1.1 Comparative network modeling by augmented Sparse Inverse Covariance Selection.

In this section we describe the proposed model. The key novel elements of our framework, discussed in separate sections below, are:

1. Network estimation across multiple cancer data sets via the fused graphical elastic net penalty
2. Sample size correction to reduce the impact of unbalanced data sets across different cancers
3. Integration of multiple data types (mRNA, CNA, miRNA, methylation, mutation) using an informative prior
4. Improved stability and interpretation of models via modular constraints
5. Subsampling methods and cluster (parallel) computing for efficient network construction
6. Stable and robust comparative network models via bootstrap ensemble estimates

1.1.1 The fused graphical elastic net penalty.

The SICS model assumes that data X, with n observations (tumors) and p variables (mRNAs, miRNAs, etc), are independently distributed (across tumors) as $N(0, \Sigma)$, where Σ is the $p \times p$ correlation matrix. The network is represented by the *precision matrix* $\Theta = \Sigma^{-1}$, whose non-zero elements capture direct dependencies between variables. Specifically, variable i is conditionally independent of variable j given all the others (i.e. there is no link between i and j), if and only if the (i, j)-th element in the precision matrix is zero. GGMs can be extended to multiple datasets X^1, $X^2, \ldots, X^C$, where C is the number of cancer classes. Data X^c, with n_c observations (tumors) and p variables, which are common to all C datasets, are assumed to be i.i.d. $N(0, \Sigma^c)$. The cancer specific networks are represented by the precision matrices $\Theta^c = (\Sigma^c)^{-1}$, $c = 1, 2, \ldots, C$. We thus derive network models by optimizing the following penalized

likelihood function:

$$\min_{\Theta^c, c=1,\ldots,C} \sum_{c=1}^{C} \underbrace{-n_c(\log\det\Theta^c - \mathrm{tr}(S^c\Theta^c))}_{negative\ log-likelihood} \tag{1}$$

$$+ \underbrace{\sum_{c=1}^{C}\sum_{i\neq j} \lambda_1^c \nu_{ij} \left[\alpha \mid \theta_{ij}^c \mid + (1-\alpha)(\theta_{ij}^c)^2\right]}_{sparsity\ constraint} + \underbrace{\sum_{c<c'}\sum_{i\neq j} \lambda_2^{cc'} \omega_{ij}^{cc'} \mid \theta_{ij}^c - \theta_{ij}^{c'} \mid}_{differential\ connectivity\ constraint},$$

where θ_{ij}^c is the ij element of Θ^c, and S^c is the sample correlation matrix of cancer class c. The first penalty term controls the overall sparsity level of all precision matrices (1): the larger the tuning parameters λ_1^c are, the sparser the Θ^c are. The *elastic net* parameter α improves the estimation stability in the presence of highly correlated variables (2). When $\alpha = 1$ the penalty corresponds to a pure lasso penalty, and when $\alpha = 0$ the penalty corresponds to ridge regression penalty. Common choices are 0.90 and 0.95 (3; 4) Here, α is set to a value of 0.95, which produces a sparse network. Sensitivity analysis (using $\lambda_2 = 0$ and λ_1 set to produce networks of 2800-3000 links) showed that changing α in the interval 0.9 to 1.0 changes network structure by less than 5 % (Figure S1). The second penalty term is the so-called fused penalty (5; 6), where the parameter $\lambda_2^{cc'}$ controls the degree of differential connectivity, i.e. the tendency of links to be shared across cancers: the larger it is, the more the link value is constrained to a common value across the pair of cancer classes c and c'. The sparsity constraints are further augmented by prior factors, ν_{ij}, and adaptive modular factors, $\omega_{ij}^{cc'}$ (see below).

In practice, integration of data types will include both continuous (e.g. mRNA) and binary (e.g. mutation) data. The partial correlations can be obtained from the precision matrix, for any distribution (7), thus construction of networks from partial correlations is well defined for all types of data, i.e. not only continuous Gaussian distributed measurements.

Banerjee et al (8) showed that sparse maximum likelihood estimation of partial correlations for binary random variables can be well approximated by the Gaussian model. Recent work has focused on semi-parametric modeling via copula graphical models, e.g. (9) which extends graphical models to a mix of variable types. The idea behind copula graphical models is to estimate an optimal marginal transformation for each variable such that the transformed variables can be modeled with a multivariate Gaussian likelihood. In (9) the authors show that robust and efficient estimates of the partial correlation is possible without directly estimating the marginal transformations. Instead, a nonparametric estimate of variable-association, e.g. rank correlation, is used as input into the Gaussian graphical model. The strong results, both theoretical and simulation-based, of (9) motivate us to use the SICS framework to model TCGA data. For variables that exhibit non-Gaussian characteristics, rank-correlation thus serves as a proxy for marginal variable transformations.

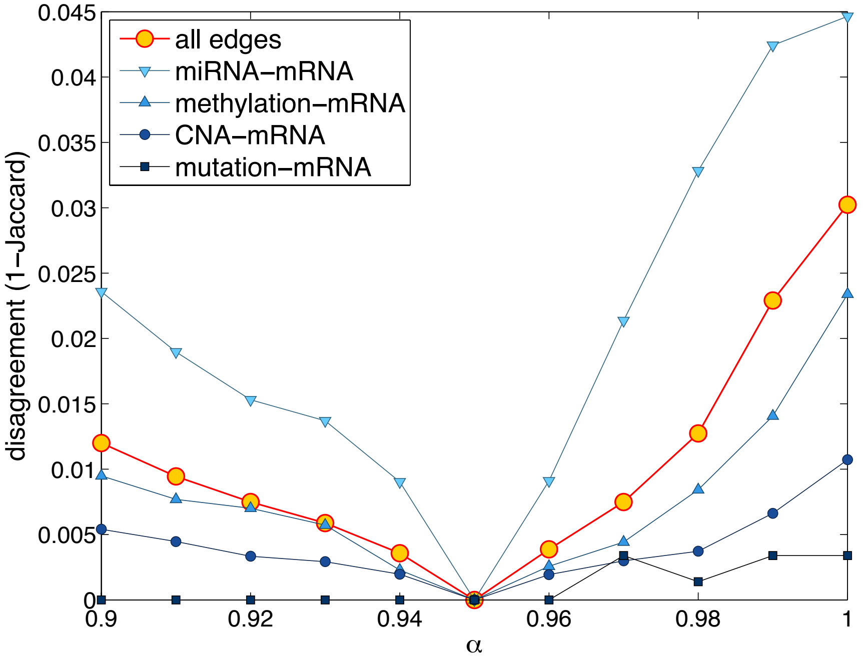

Figure S1: Sensitivity analysis with respect to parameter α. We changed α in the interval 0.9 to 1.0. Compared to the default value of 0.95, the network structure changes by less than 5%, measured by 1-Jaccard index.

1.1.2 Sample size correction to reduce the impact of unbalanced data sets across different cancers

If identical penalty parameters, λ_1 and λ_2, are used for all cancer classes, it is a simple exercise to show that the *effective* penalties are: (a) λ_1/n_c, for the sparsity constraint, where n_c is the number of cases for each cancer, resulting in more sparse networks for cancer classes with smaller sample sizes; and (b) $\lambda_2 \frac{n_c+n_{c'}}{2n_c n_{c'}}$, for the differential connectivity constraint between cancer classes c and c', resulting in a more aggressive penalty on differential connectivity between small cancer classes. This dependence on sample size is undesired, since it is biologically reasonable to assume that different cancer classes have similar network sizes. Also, if data sets with extremely small sample sizes are included, their estimates would be close to empty and/or equal, rendering them uninformative. To control this behavior we define new effective penalty parameters as $\lambda_1^c = \lambda_1 n_c^e$ and $\lambda_2^{cc'} = \lambda_2 \frac{2n_c^e n_{c'}^e}{n_c^e + n_{c'}^e}$, where $n_c^e = \bar{n}^\delta n_c^{(1-\delta)}$, $\bar{n} = \frac{1}{C}\sum_{c=1}^{c} n_c$ and δ is a parameter between 0 and 1 which controls the degree of sample size correction. To set a value for δ, we solve the SICS optimization problem for different values of δ and λ_1, to identify a point where the networks for the different cancers have similar size, measured by the maximum pairwise difference in network size between any two cancers (Figure S2).

To test our sample size correction proposal, we set up a simulation study as follows. We simulate a data set with $C = 3$ classes and $p = 250$ variables each. We subsequently construct networks for different values

of δ and λ_1 (Figure S2a), and compare the estimated networks to the true network and compute the true positive rate (TPR, proportion of true links found in the estimated network) constraining the false positive rate (FPR, proportion of false links found in the estimated network) to some small value. The maximum TPR is achieved for $\delta \simeq 0.4$ across a wide range of FPR constraints (Figure S2b). This illustrates that sample size correction is motivated since otherwise $\delta = 1$ would have yielded a higher TPR. It also illustrates that no correction (corresponding to $\delta = 1$), or naive sample size correction (6), (corresponding to $\delta = 0$), is not optimal. For the TCGA data, we choose δ as the value that minimizes the maximum pairwise difference of network sizes for the different cancers. That is, we assume that an appropriate sample size correction should result in network sizes of similar size across all cancers. The maximum pairwise difference criterion is supported by results from the controlled simulation setting showing that the maximum pairwise difference measure is optimized near the optimum for TPR. By this criterion, for TCGA data δ should be in the range 0.05-0.1 (Figure S2c), and for our analyses we chose to use $\delta = 0.08$. Lower values of δ will make augmented SICS detect more links for cancers with a high number of patients, and higher values of δ will make the method detect more links for cancers with a low number of patients.

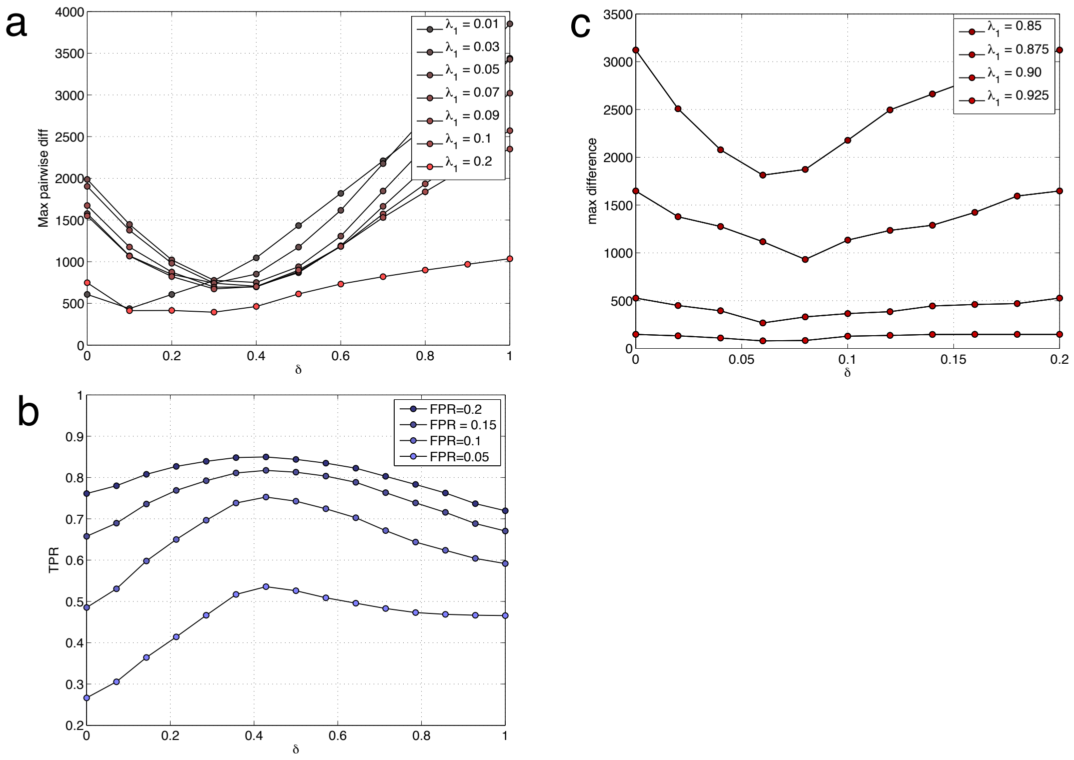

Figure S2: Choice of the sample size correction parameter δ. See text for details. (a) simulated data for three cancers: changing the optimization parameters δ and λ_1 find a point where the maximum pairwise network difference is small ($\delta \approx 0.4$). (b) selecting δ to produce a similar network size for the different cancers maximizes the true positive rate (TPR) of detected link. (c) curves used to select δ for the TCGA data.

1.1.3 Prior to facilitate detection of miRNA targets, *cis* methylation effects and impact of mutations.

The global objective function includes a *link specific prior*, ν_{ij}, which is designed to tune the sparsity penalty for forming an link between network nodes i and j. The sparsity penalty for link element (i, j) is defined as $\lambda_{1,ij} = \lambda_1 * \nu_{ij}$, where λ_1 is a common factor that controls the overall sparsity of the network, and ν_{ij} take on three possible values: 1, u (< 1), or ∞. The motivation for this choice of prior is that it can serve to emphasize features of the model that are either more likely, based on prior information, or are of higher biological interest to the end user. In such cases ν_{ij} is set to the value $u < 1$. This reduced penalty is applied in the following situations:

- between miRNAs with their predicted mRNA targets, as defined by miRanda (10) prediction (MicroCosm Targets Version 5 (11), http://www.ebi.ac.uk/enright-srv/microcosm/htdocs/targets/v5/). This choice is motivated by the belief that such links are more likely to be real than links that do not involve defined miRNA-mRNA target relationships.
- between *cis* localized methylations probes with their corresponding mRNA, as defined by associations between genes and methylation probes provided in the level 3 data provided by TCGA. This choice is motivated by the belief that such *cis*-localized probes are likely to be involved in transcriptional suppression. Many of the detected links between promoter methylations and mRNAs do indeed have a negative sign, consistent with this expectation (Figure 2).
- between all interactions involving a point mutation. This choice is motivated by the belief that point mutations are key determinants of the molecular phenotype.

In addition, the prior is used to model the assumption that the effect of CNAs on transcription is only via *cis*-effects, i.e. mRNAs can only be linked to CNAs at their coding locus, which is done by setting $\nu_{ij} = \infty$ for all trans-interactions that involved CNA and an mRNA, and $\nu_{ij} = 1$ for all *cis* interactions. We chose the value $u = 0.75$ in our analyses, and found upon inspection that this prior helps give a balanced model, with involvement of the different data types.

With the proposed prior model, we primarily seek to obtain a well balanced model with connection between the data types. Using no prior at all produced results with an extensive number of links produced between CNAs in close genetic proximity and methylation probes in close genetic proximity, which we regard as a less informative network. We therefore also set $\nu_{ij} = \infty$ for such connections. We performed a simulation study to investigate the impact of this last restriction and found that while the network weights for other network connections were altered, the network structure itself was not much affected. Also, we investigated the enrichment of variable pairs included in the prior among strong correlations compared to weak or opposite sign correlations (Supplementary Table 1). This shows a significant enrichment for target predicted

miRNA-mRNA associations for correlations < -0.2 compared to correlations ≥ -0.2. It also displays significant enrichment for *cis* located methylation probes and mRNA among strong negative correlations, and for *cis* interactions between CNA and mRNA among strong positive correlations.

To quantify the overall impact that the prior has on the network we performed a sensitivity analysis, in which we explore the effect of changing the prior strength u (defined above) from its default value of 0.75 to values in the range 0.50 (stronger prior) to 1.00 (a flat prior). When increasing u to 1.0, less than 4% of links changed for the whole network, and up to 15% of links changed for miRNA-mRNA links (Figure S3). When instead reducing the prior, a similar effect is seen: fewer than 10% of links changed for the whole network, and up to 35% of links changed for miRNA-mRNA links. For the case of a flat prior (no link specific information provided), we further explored if a network built using a flat prior would enrich for the link-specific prior. Such a simulation showed up to 100-fold enrichment of our prior links. The enrichment depended on the data type: for CNA-mRNA and DNA methylation-mRNA links the enrichment was up to 100-fold, and for miRNA-mRNA links, the enrichment was up to 5-fold (Figure S4). The simulations in this test were done using $\lambda_2 = 0$ and λ_1 set to produce networks of 2800-3000 links.

In summary, while a prior formally does not require validation (because it reflects a belief), our assessment of the prior shows that it is informative and has a moderate and tunable effect on the network solution. Changing the prior structure will likely be useful to bring forward different aspects of the data, reserved for future work.

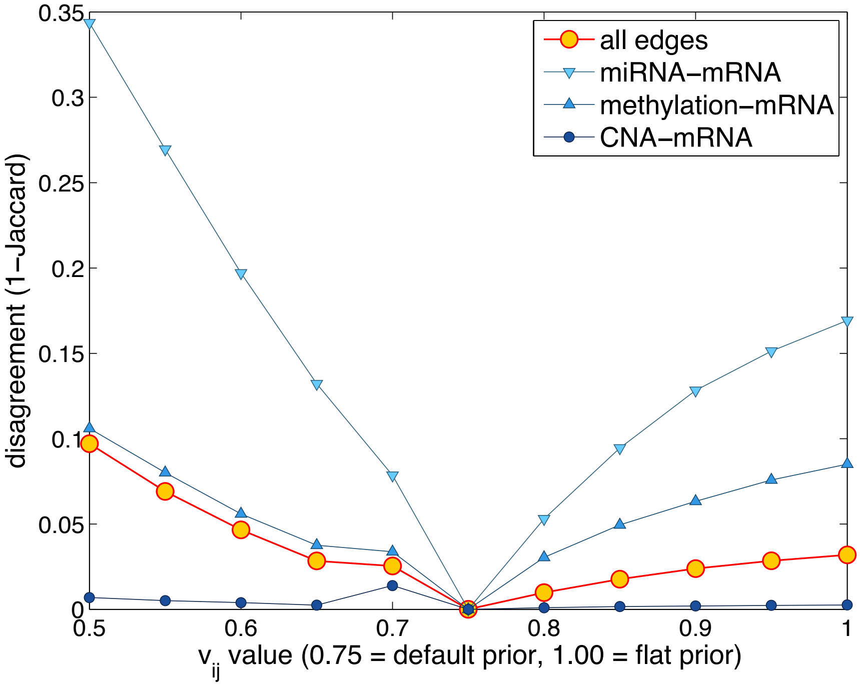

Figure S3: Effect of tuning the prior strength u. Networks constructed using the default value of u=0.75 were compared to networks constructed for a range of u values between 0.50 (a stronger prior) and 1.00 (a flat prior). The average network difference (1-Jaccard index, averaged across the 8 cancers) was used to quantify the deviation from the u=0.75 case. The analysis showed that the effect of the prior is moderate, changing up to 10% of the entire network structure. miRNA-mRNA links changed less than 20% when comparing our default value to a flat prior, indicating that such links are to a high degree determined by the data rather than the prior.

1.1.4 Improved stability and interpretation of models via modular constraints.

The adaptive factor $\omega_{ij}^{cc'}$ is designed to improve the stability of the network estimates and generate interpretable networks. This is done by a two-step adaptive lasso (12) method, in which preliminary network estimates (obtained using $\omega_{ij}^{cc'} = 1$) are used to update $\omega_{ij}^{cc'}$ to a new value obtained from the initial network estimate $\tilde{\Theta}$. The purpose of the update is to encourage all links within a module, or local sub-network, to exhibit the same link commonality or link differential connectivity properties across cancers. This is achieved by the following update:

$$\omega_{ij}^{cc'} = \left[\mid \tilde{\theta}_{ij}^{c} - \tilde{\theta}_{ij}^{c'} \mid \sum_{l \in N_{ij}} \left(\mid \tilde{\theta}_{il}^{c} - \tilde{\theta}_{il}^{c'} \mid + \mid \tilde{\theta}_{jl}^{c} - \tilde{\theta}_{jl}^{c'} \mid \right) \right]^{-1}.$$

N_{ij} denotes the set of neighbors of link (i, j), i.e. the set of links connected to nodes i and/or j. The

mRNA + miRNA<br>t	-0.6	-0.5	-0.4	-0.3	-0.2	-0.1
gbm	i.d.	0.322931	0.110545	0.00212674	4.27073e-05	0.000268926
ov	i.d.	i.d.	0.0386176	0.0475301	0.00148755	3.86216e-16
brca	i.d.	0.712427	0.613157	0.149858	0.000522543	1.48539e-05
hnsc	i.d.	0.866089	0.213696	0.675552	0.00194067	3.12862e-06
ucec	i.d.	0.446383	0.0659011	0.00844801	1.845e-09	5.71009e-06
kirc	0.0579481	1	0.00403357	5.66623e-05	8.88601e-08	0.00590488
lusc	i.d.	0.805025	0.130049	0.013695	0.00069983	7.59039e-05
mRNA + methylation<br>t	-0.8	-0.7	-0.6	-0.5	-0.4	-0.3
gbm	i.d.	0.000116723	1.55846e-15	3.86981e-49	5.08427e-100	3.30879e-169
ov	5.83631e-05	2.3827e-11	1.59542e-68	5.85689e-125	9.93166e-277	0
brca	i.d.	4.75078e-50	3.35902e-126	6.66071e-269	0	0
hnsc	i.d.	1.08439e-19	3.02118e-99	4.46074e-200	1.59075e-288	0
ucec	3.40522e-09	1.71652e-49	2.22401e-139	6.2202e-294	0	0
kirc	i.d.	1.81471e-13	1.26672e-70	7.23213e-163	9.46097e-309	0
lusc	1.15815e-17	3.23378e-66	1.93972e-138	6.46009e-203	2.76041e-299	1.40629e-284
coad	4.67951e-11	2.41947e-39	1.85133e-74	1.80665e-128	1.98331e-152	6.49899e-136
mRNA + CNA<br>t	0.4	0.5	0.6	0.7	0.8	0.9
GBM	0	0	2.2628e-250	4.45682e-103	6.38925e-36	i.d.
OV	0	0	0	0	2.41511e-23	i.d.
BRCA	0	0	0	0	0	3.15044e-22
HNSC	0	0	0	0	6.27697e-255	9.20421e-17
UCEC	0	0	0	2.52093e-171	5.1871e-14	i.d.
KIRC	0	0	3.0966e-220	7.70777e-26	i.d.	i.d.
LUSC	0	0	0	0	2.15214e-211	1.09726e-10
COAD	0	0	0	9.21342e-279	1.57279e-60	i.d.

Table S1: Enrichment of variable pairs included in prior among strong correlations. mRNA + miRNA: Enrichment of miRNA targets among correlations $< t$ compared to correlations $\geq t$. **mRNA + methylation:** Enrichment of interactions between *cis* located methylation probes and mRNA among correlations $< t$ compared to correlations $\geq t$, all methylation-mRNA associations included. **mRNA + CNA:** Enrichment of *cis* interactions between CNA and mRNA among correlations $> t$ compared to correlations $\leq t$, all CNA-mRNA associations included. Fisher's exact test was used for all enrichment calculations, i.d.= insufficient data (no observations above/below threshold).

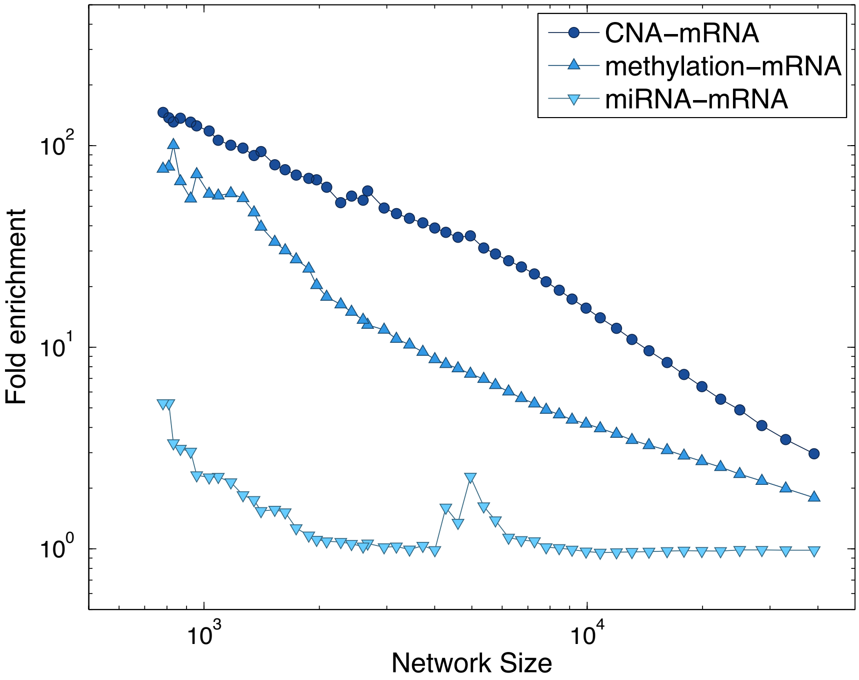

Figure S4: Constructing networks using a flat (non-informative) prior enriches for prior links. The presented method uses link-specific priors in three different contexts: CNA-mRNA links (penalty = inf for different locus), methylation-mRNA (reduced penalty for methylation in the same (cis) promoter) and miRNA-mRNA (reduced penalty for miRNA-mRNA pairs for which there is a miRanda target prediction. We used the same fold enrichment statistic as in the main manuscript (c.f. Figure 2b). Curves are the average for 8 TCGA cancers. Similar to our analysis of PathwayCommons links, our method enriches also for priors by a factor ranging from 5 (miRNA links) to >100 (CNA-mRNA links).

adaptivity factor $\omega_{ij}^{cc'}$ encourages fusing of link (i, j) for cancer classes c and c' when (a) their link values, $\tilde{\theta}_{ij}^{c}$ and $\tilde{\theta}_{ij}^{c'}$, are close in the initial network estimate and/or (b) when this is true for neighboring links.

Applying this update to the TCGA data, we note improvement in network estimation stability, measured by Kendall's W (Figure S5a) and enrichment of links from PathwayCommons (Figure S5b). These metrics, previously used in (13) are described below.

1.1.5 Robust network construction using bootstrap.

Network estimation is a difficult problem and the results are often unstable. To produce robust network estimates we propose to use the bootstrap and compute aggregated results. Specifically, we repeat the estimation on $B = 500$ (Figure S6) bootstrap data sets which consist of 90% randomly chosen tumors from each cancer class. Pseudo-code for the construction of a correlation matrix based on bootstrapped data is

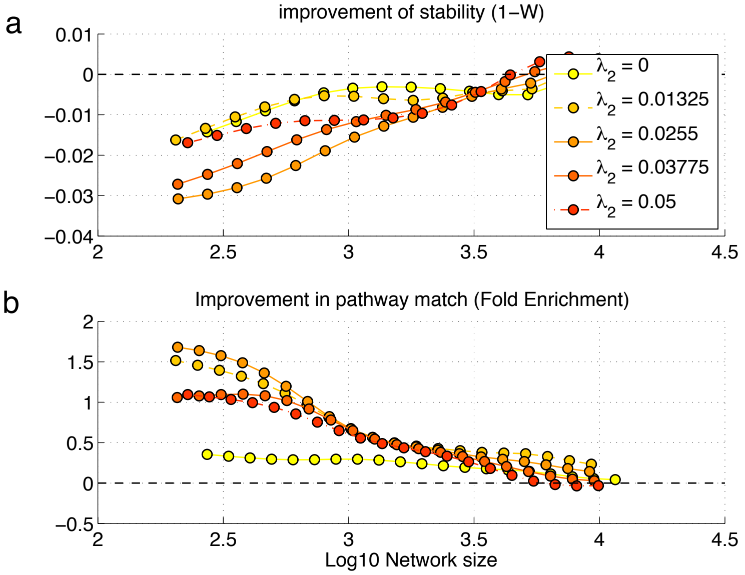

Figure S5: modular constraints on network structure improves stability and functional enrichment. We derived networks from TCGA data with (curves) and without (baseline, y=0) modular constraints. The y axis represents the improvement of network robustness, measured as 1-Kendall's W (a) and pathway enrichment, defined below (b). The x axis represents the logarithm base 10 of network size.

available in section 1.3.2. For each bootstrap sample b we estimate the corresponding network and compute two frequency statistics; (1) the proportion of bootstrap networks where link (i, j) is present, given by

$$n_{ij}^c = \sum_{b=1}^{B} I\{\theta_{ij,b}^c \neq 0\}/B,$$

where $\theta_{ij,b}^c$ is the unsigned link estimate of the b-th bootstrap network for cancer class c; and (2), an estimate of the probability that link (i, j) differs in value between cancer classes c and c' given the link is present in both

$$n_{ij}^{cc'} = \frac{\sum_{b=1}^{B} I\{\theta_{ijb}^c \neq \theta_{ijb}^{c'}, \theta_{ij}^c \neq 0, \theta_{ij}^{c'} \neq 0\}}{\sum_{b=1}^{B} I\{\theta_{ij}^c \neq 0, \theta_{ij}^{c'} \neq 0\}}.$$

Links whose frequency statistics n_{ij}^c and $n_{ij}^{cc'}$ exceed a threshold T_p and T_f, respectively, constitute our (unsigned) network estimate. Specifically, the threshold T_p thus controls the stability of the sparsity structure of the estimates: its value corresponds to the minimum proportion of bootstrap estimates in which an link is present. The final differential connectivity pattern is obtained using $n_{ij}^{cc'}$ as input into hierarchical clustering, then cutting the resulting cluster tree at height T_f. The threshold T_f controls the robustness of the differential connectivity pattern since its value corresponds to the maximum proportion of bootstrap estimates below which a link is fused.

Finally, we estimate the link signs. For differential links, let N_+ and N_- be the number of bootstrap estimates in which the links were positive and negative, respectively. If $N_- < N_+$, the final estimate for the link is negative, otherwise is positive. For links whose values coincide across cancers, we similarly, define $N_+^{c_i}$ and $N_-^{c_i}$ for cancer c_i. Let $N_+ = \sum_{c_1}^{c_f} N_+^{c_i}$, and $N_- = \sum_{c_1}^{c_f} N_-^{c_i}$, where the sum runs for the cancers in which the links are fused. If $N_- < N_+$, let the final estimate for the link be negative, otherwise positive.

The bootstrap link frequency histograms are highly informative. Reasonable values for the penalty parameters, λ_1 and λ_2, result in bootstrap frequency histograms with a distinct U-shape (see Figure 2a in main manuscript and Figure S7a), indicating that the links comprise two populations: those frequently present (or differential) and therefore likely to correspond to "true" findings, and those frequently absent (common) in the network estimate. If λ_1 and/or λ_2 are too small or too large, the histograms shift toward the right or left, suggesting an overfit or underfit of the network models. Given a U-shaped histogram, the thresholds T_p and T_f should be set at values that separate the two link populations seen in the histograms. In Cancer Landscapes we allow for exploration of networks of different sizes (controlled by the penalty parameter λ_1). The final networks presented result from a threshold value of $T_p = 80\%$. Similarly, the presented cancer specific differential connectivity patterns result from a threshold value $T_f = 60\%$. The results are quite robust with respect to the threshold value used (see below). These particular values were chosen for the presented networks from a simulation study, based on preliminary estimated networks, where the FDR with respect to the known simulation-model was computed.

1.1.6 Estimation of FDR from bootstrap data.

The FDR with respect to T_p and λ_1 is defined as the proportion of false links present in the estimated network. The FDR with respect to T_f and λ_2 is defined as the proportion of false differential links among links estimated to be differential. In the simulation study that was the basis for selecting a bootstrap threshold, the true and false links (in terms of presence and/or differential presence between cancers) were known and the FDR could be directly computed. The bootstrap data can also be used to *estimate* the FDR when the true network model is not known.

Building on the BINCO procedure (14), we estimate FDR as follows. For a bootstrap frequency histogram, we model the two populations of links using a mixture of beta-binomials (see Figure 2a, Figure S7). This allows for an link-specific inclusion probability in the network estimate. We extend the BINCO-procedure for the false links (that should not be present in the models) using a zero-inflated beta-binomial to take the high-dimensionality and extreme sparseness of TCGA large-scale network models into account (15). Once the mixture model is fit to the bootstrap link frequency histograms, we obtain a model-based estimate of the

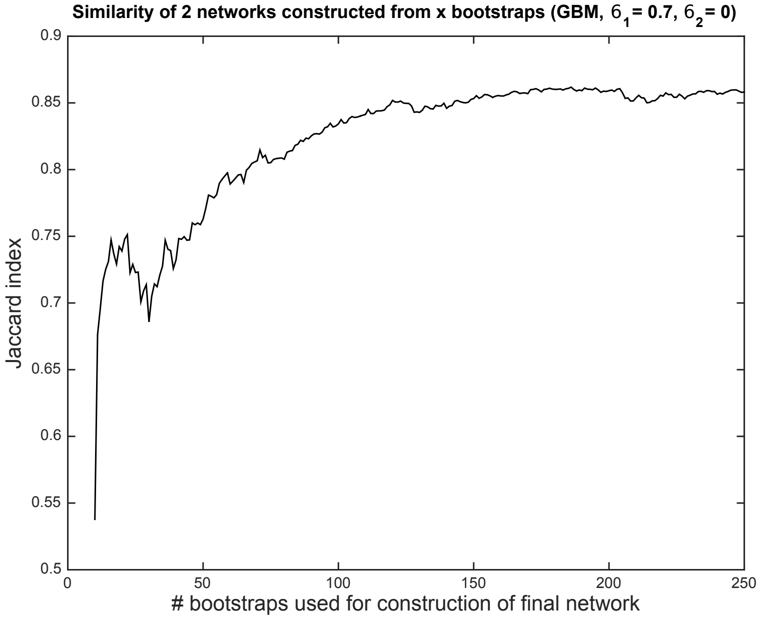

Figure S6: Investigation of the effect of number of bootstrap data sets on network stability. We investigated how the number of bootstraps affected the final network by comparing two networks constructed from 10 to 250 bootstrap data sets (x-axis). The two networks were compared using Jaccard index (y-axis), which stabilize around 0.85 already at 200 bootstraps showing that 500 is a sufficient number of bootstrap data sets.

FDR as a function of the bootstrap threshold:

$$FDR_{detection}(T_p) = \frac{Prob(link\ freq.\ count > T_p \mid link\ is\ false)}{Prob(link\ freq.\ count > T_p)}$$

where the numerator is the area under the estimated density above threshold T_p for the false link population and the denominator is the sum of this and the area under the estimated density above threshold T_p for the true link population.

Recognizing that a similar FDR estimation procedure can be applied to the differential connectivity problem, we fit a beta-binomial mixture to each of the pairwise cancer comparison frequency histograms (Figure S7). We can thus estimate the FDR for detecting differential connectivity for each cancer comparison as

$$FDR_{differential}(T_f) = \frac{Prob(link\ differential\ freq.\ count > T_f \mid link\ false\ differential)}{Prob(link\ differential\ freq.\ count > T_f)}$$

Figure S7a illustrates the procedure. The red line is the false differential link density and the blue the true differential link density. FDR for a vertical cut, T, along the x-axis correspond to the area under the red curve to the right of T divided by the sum of this and the area under the blue curve to the right of T (14).

In Figure S7b we summarize our findings. The estimated FDR is clearly quite robust and not much affected by the particular threshold used. This is due to the stability of the estimates across bootstraps. The histograms indicate that most links are persistently differential or common (extreme U-shape of the histograms). Moreover, the FDR for differential connectivity is controlled below 10% for reasonable values of the fuse penalty, λ_2. For values of λ_2 exceeding 0.0075 FDR increases and cannot be controlled below 10%, suggesting an over-penalized model. We can clearly see that the FDR is minimized for λ_2 between 0.0025 and 0.005, indicating that these values for the tuning parameter produce the most stable differential connectivity estimates across bootstraps. These are the penalty levels used to produce the networks analyzed in the main manuscript.

There is a complex relationship between penalties λ_1 and λ_2. Large λ_1 result in sparse networks. For such estimate we can control the FDR for differential connectivity for small values of λ_2, suggesting that small networks are dominated by differential links (cancer specificity). For example, FDR<10% for $\lambda_1 = 0.7$ when $\lambda_2 < 0.015$, for $\lambda_1 = .8$ when $\lambda_2 < 0.0075$, and for $\lambda_1 = .9$ when $\lambda_2 < 0.005$.

Subsampling and cluster computing.

Estimation of a network on all variables (more than 1 billion pairs of variables) was practically unfeasible for further analysis. We therefore first filter the variables as follows. Only pairs of variables with correlation above the threshold 0.7, in any cancer, are considered. This screening method is valid for values of the sparsity penalty $\lambda_1 \geq 0.7$ since variables with correlations < 0.7 will by construction not be part of the estimated networks (6). The screening thus accelerates computation time by dropping irrelevant variables without affecting network quality. To account for the prior (Section 4 above), CNA or methylation variables correlating over the threshold but only with other CNA or methylation variables, respectively, were not included. Finally, miRNA and mutation variables were included if they correlated more than a lower threshold of $0.7 \cdot 0.75$ with any other variable. After filtering, the number of variables in our network model has been reduced to p =22,447. Pseudocode describing the filter procedure is available below in section 1.3.3.

To further reduce the execution time, we adopt the blocking method of the correlation matrices by (1; 6) and implement a subsampling method for each bootstrap as follows. Blocking of the correlation matrices into subproblems was done for each cancer separately, for $\lambda_2 = 0$ and the lowest chosen setting of λ_1. Each of the subproblems with number of variables ≥ 1000 was subject to a subsampling procedure:

All variables included in the union of all V^r, or present in a subproblem with number of variables less than 1000, were included in the next step. The union of selected variables over cancers was used for further analysis. We performed several simulation studies to decide on the number of subsampling steps and the

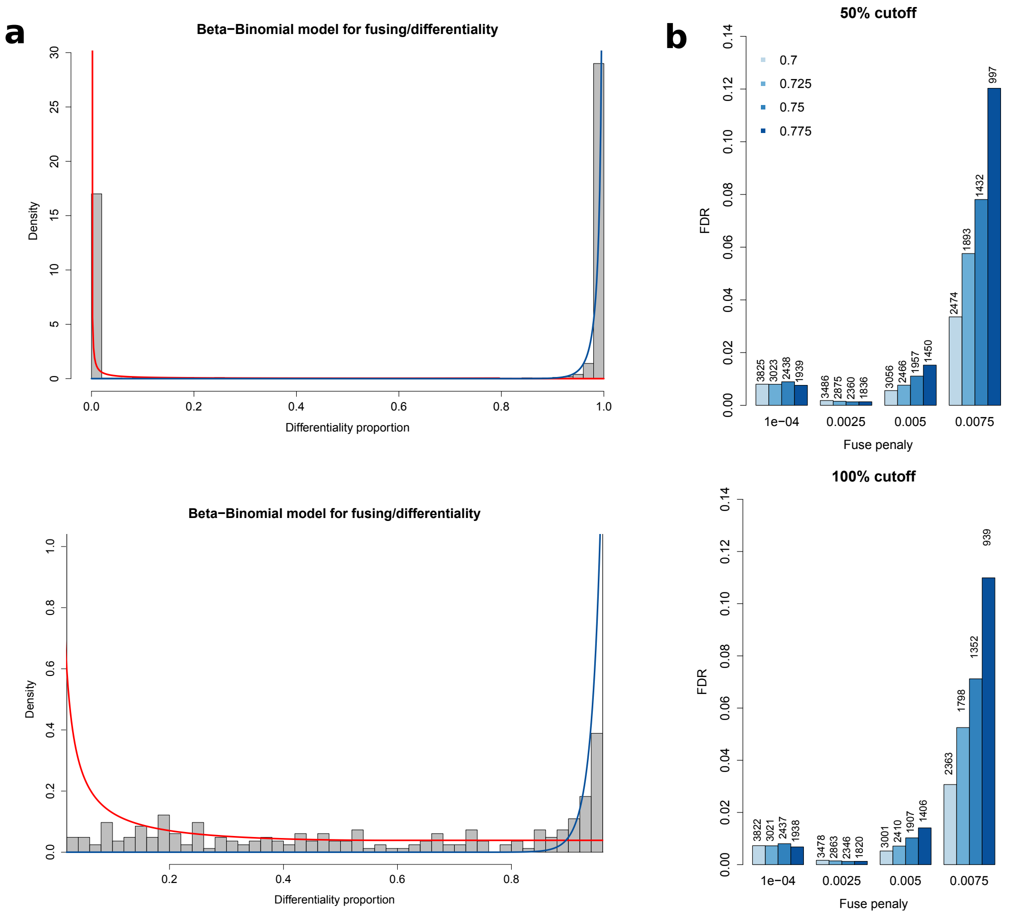

Figure S7: a: Beta binomial mixture distribution to estimate FDR of differential network connectivity. Bars represent the fraction of bootstrap simulation in which two network links are differential between a pair of cancers. Red and blue curves: estimated probability density function (mixture beta-binomial) of false and true differential links, respectively. Bottom panel is a detailed view of the histogram and model fit, excluding the dominant 0 and 100% counts. This detailed view is provided for illustrative purposes since it clearly shows that there are differential frequency counts in the entire range of 0 to 100%. We also see that the false differential link distribution (red line) is flat toward the right side of the histogram, which explains the stability of the FDR estimate as a function of bootstrap threshold (panel b). **b: FDR estimates of differential connectivity** computed at two different cut-offs (50% of bootstrap runs, and 100%, respectively). Bars represent FDR values obtained for different fuse penalties (λ_2 value, groups along X axis) and different sparsity penalties (λ_1 value, blue shades). Numbers over bars indicate network size. Note that FDR is well below 5% for fuse penalties less than 0.0075.

size of the subsampling problem. With the above setting we are ensured that all variables get a chance to "compete" to be part of the final network model.

We optimize the penalized likelihood using the *alternating directions method of multipliers*, ADMM. For a complete description of the method see (16) and (17). To speed up the estimation, link-weight updates in the

for $r = 1 \to 100$ **do**
$S^r \leftarrow$ correlation matrix for 100 randomly picked variables
$\hat{\Theta}^r \leftarrow$ estimated network from S^r
$V^r \leftarrow$ variables present in $\hat{\Theta}^r$
end for

iterative algorithm are vectorized. In order to enable simulations for multiple penalty parameter settings, we set up a computational framework to interact with a 268 node computer cluster (C3SE) localized at Chalmers University of Technology in Gothenburg, Sweden. The implementation of our method and loading of the data from a mySQL database was done in Matlab.

Source code in Matlab is available as Supplementary files. The main script for the ADMM solver is named codeMatlab/ADMMk/ADMMk.m. Scripts, and data matrices (codeMatlab/cancerdata.tar.gz) are also available for calculation of the correlation matrix S and the prior matrix L, main scripts codeMatlab/prepare_S_Lp/computeSmatrix.m and codeMatlab/prepare_S_Lp/constructLprior.m. Additionally, the Matlab scripts used to calculate all bootstrapped networks and summarize them are given, main script CodeMatlab/cluster/Main.m. These scripts, however, are specifically designed for the C3SE cluster and requires adaption to a user specific environment.

1.2 Analysis of method performance (estimation robustness)

1.2.1 Performance metrics.

We used two different metrics to characterize performance, previously described in (13).

For analyses in Figure 2b and Figure S5b, estimated networks were compared against pathway databases HPRD, NCI-NATURE, REACTOME and IntAct downloaded from Pathwaycommons.org. We map identifiers in the databases to our set of variables. We then compute the shortest path, P_{ij} from gene pairs (i, j) in the database using Johnson's algorithm. We define the pathway enrichment of a network Θ as follows:

$$\frac{Prob(P_{ij} = k \mid \text{link } (i,j) \text{ present in } \Theta)}{Prob(P_{ij} = k \mid \text{link } (i,j) \text{ present in } \Theta_{\text{permuted}})},$$

The graph Θ_{permuted} is obtained by randomly permuting the rows in Θ (and the columns the same way), equivalent to randomly re-assigning gene names. We used a path-length $k = 2$ in our calculations. The

numerator and denominator were estimated as follows:

$$Prob(P_{ij} = k \mid \text{link } (i,j) \text{ present in } \Theta) = \sum_{i,j} I\{P_{ij} = k \mid \theta_{ij} \neq 0\}/N$$

where $N = p * (p-1)/2$ is the total number of possible links in the network. Similarly,

$$Prob(P_{ij} = k \mid \text{link } (i,j) \text{ present in } \Theta_{\text{permuted}}) = \sum_{r=1}^{R} \sum_{i,j} I\{P_{ij} = k \mid \theta_{ij,permuted} \neq 0\}/NR$$

where $R = 100$ is the number of random permutation graphs created.

For the analysis of estimation stability (robustness) of networks, we used 1-W, where W=Kendall's correlation coefficient, plotted as a function of network size (c.f. (13)). A low value of 1-W indicates that the method gives comparably stable network structures.

1.2.2 Methods analyzed

Using the two above metrics (enrichment and stability) we compared the proposed augmented SICS to the following methods:

- **glasso** (1) – a partial correlation based method. It estimates the precision matrix (inverse correlation matrix) by optimizing its lasso penalized likelihood function. The lasso penalty introduces sparsity in the precision matrix by penalizing the absolute value of its elements. A penalty parameter λ controls the sparsity degree.
- **WGCNA** (18) – a correlation based network model. The absolute values of elements of the correlation matrix are raised to a power $\beta \geq 1$. The role of β is to down-weight the links with low correlation in the graph. For high values of β, small correlation values are reduced to almost 0 weight. A threshold on the link weights S_{ij}^{β} at e.g. 10^{-4} produces a sparse graph. The degree of sparsity is thus controlled by the power β.
- **ARACNE** (19) – a method that computes pairwise connectivity strengths between variables using mutual information. To introduce sparsity, the number of false positive links in this graph is reduced by considering triplets of variables for which all three pairwise strengths exceed a significance threshold (i.e. were part of the sparse graph). Using the data processing inequality (DPI), the smallest link among the triplets is removed.

- **MINE** (20) establishes the dependence strength of a pair of variables through the maximal information coefficient (MIC). The MIC is built on the fact that a data scatterplot can be partitioned by a grid that best encloses their relationship, if existent. It is computed as the maximum mutual information that the data can achieve given a certain grid. Sparsity is introduced by multiple testing on the estimated MIC scores.

In a comparison that focused on all mRNA data (9104 mRNA variables that passed the filter described above), we saw strong performance of the proposed SICS method in terms of pathway enrichment scores (Figure 2, main paper). This comparison could not include MINE because of prohibitively long run times. To include MINE, we conducted a more limited analysis as follows. On the three cancers with the largest number of tumors in the TCGA database (glioblastoma, breast cancer and ovarian cancer), we selected a random subset of 500 mRNA transcripts from the 1103 transcripts that passed the variable filtering step described above. We then randomly split the tumors into two datasets per cancer. For each dataset we estimate networks using all methods, and sweep over tuning parameter values that control the sparsity of the network. For SICS this corresponds to varying λ, for ARACNE DPI, for WGCNA β, for MINE the MIC scores, and for augmented SICS λ_1 and λ_2. We consider each λ_2 setting as a separate method, with different levels of stringency applied to differential link connectivity. All the methods thus generate a series of networks with different sparsity levels. The entire procedure is repeated $B = 10$ times. For fairness of comparison, we did not use the bootstrap robust estimation for our method here.

We depict $1 - W$ as a function of network size in Figure S8. Thus, the network sizes that are deemed stable correspond to minimum values in the figures. Results show that augmented SICS with higher λ_2, as well as WGCNA, are both stable methods. However, augmented SICS networks are most stable for network with size around 1000 links whereas WGCNA favor networks with 3000 links.. This result is consistent, since the correlation based network corresponding to a sparse partial correlation one is expected to be denser. ARACNE performs poorly. Augmented SICS networks with moderate λ_2 outperforms standard SICS. MINE performs similarly to WGCNA, but is computationally more heavy to apply and is not suitable for application on the full dataset.

1.3 Data preparation and processing

The proposed method can be applied to, but is not restricted to, data sets from the TCGA (the Cancer Genome Atlas,
http://cancergenome.nih.gov) database. TCGA data are organized into technical platforms, and we chose the platform for each data type and cancer that maximized the number of patients in that dataset. The number of patients available for each combination of data types is displayed in Table S2. All measurements

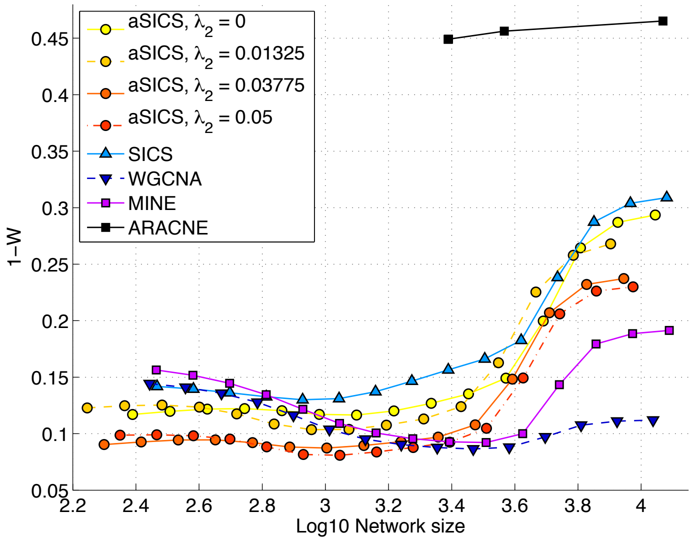

Figure S8: Stability of augmented SICS (aSICS) and a set of reference methods, measured by 1-W, where W=Kendall's correlation coefficient, plotted as a function of network size (c.f. (13)). A low value of 1-W indicates that the method gives comparably stable network structures.

were downloaded as TCGA level 3 data, except for point mutations that were downloaded as level 2 data, and were post-processed as described below. The data was assembled in a mySQL database to enable fast creation of data matrices during simulations. The following subsections contains pseudocode describing the assembling of data, construction of the bootstrapped correlation matrices, and filtering of variables.

- **mRNA and miRNA expression data.** The level 3 mRNA data provided by TCGA is a list of known protein-coding genes with their corresponding measured mRNA expression values, for each patient. Data from the Illumina RNA Sequencing platforms were log transformed. Further, all data were quantile normalized within each cancer and platform. MicroCosm Targets Version 5 (11), a summary database of published predicted miRNA targets, was used to map the miRNAs to their target genes.

- **DNA sequencing data (non synonymous somatic variants and indels)** Each gene was flagged as mutated (1) if it had at least one non-synonymous mutation called by TCGA (level 2 data). Synonymous mutations were not considered. To avoid spurious high correlations between genes with few calls, genes with fewer than 5 patients affected were not included in the analysis.

- **DNA copy number aberration data.** The level 3 CNA (genetic copy number aberration) information provided by TCGA is represented, for each patient, by the amplitude and genetic positions of beginning and end of DNA segments that have gained or lost copies. Each gene available in NCBI human Build 36.1 was mapped to the segments and assigned the amplitude of the corresponding segment. In the case of multiple segments covering the gene, the average amplitude was used, weighted

proportionally to the length of the parts of the segments covering the gene. Genes with a CNA value, but lacking a mRNA measurement were discarded from the analysis.

- **DNA Methylation data.** Methylation data in TCGA is supplied as a methylation probe with genetic position and a beta value (the ratio of the methylated probe intensity and the sum of methylated and unmethylated probe intensities). Methylation probes with a standard deviation across the patients > 0.05 were kept for further analysis. The predictions of methylation site gene targets provided by TCGA were used. The methylation probe values were replaced by their rank values in the correlation calculations, to overcome the issue of the values not following a normal distribution.

1.3.1 Preparation of data

Download data files from http://cancergenome.nih.gov, file URLs specified
in Supplementary file URLsToTCGA.csv

for $d \in \{mRNA, CNA, miRNA, methylation, mutation\}$ **do**
  if $d = mRNA$ or $d = miRNA$ **then**
    for all cancers c **do**
      $D_d^c \leftarrow$ import data ▷ matrix of size (#genes $\times$#patients)
      ▷ or (#miRNA $\times$#patients)
      if platform = "Illumina RNA Sequencing" **then**
        $D_d^c = log(D_d^c)$ ▷ Natural logarithm, elementwise
      end if
      $D_d^c = quantilenorm(D_d^d)$ ▷ Quantile normalize each array
      $D_d^c \leftarrow$ sortrows(D_d^c) ▷ sort rows in alphabetical gene or miRNA order
      Remove rows from D_d^c with variables not included in all cancers.
    end for
  else if $d = CNA$ **then**
    Map the genomic position of the genes in D_{mRNA}^c and NCBI human Build 36.1 to
    the positions of the segments contained in CNA data files
    for all cancers c **do**
      $D_{CNA}^c \leftarrow$ Assign segment amplitude to mapped genes. ▷ matrix of size
      ▷ (#genes $\times$#patients)
      $D_{CNA}^c \leftarrow$ sortrows(D_{CNA}^c) ▷ sort rows in alphabetical gene or miRNA order
      Remove rows from D_{CNA}^c with variables not included in all cancers.
    end for
  else if $d = methylation$ **then**
    for all cancers c **do**
      $D_{meth}^c \leftarrow$ import data ▷ matrix of size (#methylation probes $\times$#patients)
      Remove rows of D_{meth}^c with $>= 10\%$ missing data
      $sd \leftarrow std(D_{meth}^c)$ ▷ Standard deviation of each row
      Remove rows of D_{meth}^c with sd $<= 0.05$
      $D_{meth}^c \leftarrow knnimpute(D_{meth}^c, 5)$ ▷ Impute missing data on each row.
      $D_{meth}^c = tiedrank(D_{meth}^c)$ ▷ Compute rank of values, for each row
      $D_{meth}^c \leftarrow$ sortrows(D_{meth}^c) ▷ sort rows in alphabetical gene or miRNA order
      Remove rows from D_{meth}^c with variables not included in all cancers.
    end for

---

else if $d = mutation$ **then**
for all cancers c **do**
$D^c_{mut} \leftarrow \begin{cases} 1, & \text{if patient has at least one non-synonomous mutation call for gene} \\ 0, & \text{otherwise.} \end{cases}$ ▷matrix of size (#mutated genes ×#patients)
$N \leftarrow \#rowsum(D_{mut})$ ▷ Calculate sum of each row
$pt \leftarrow prctile(N, 99)$ ▷ Calculate 99% percentile
Remove rows of D^c_{mut} with $rowsum < pt$.
end for
end if
end for

1.3.2 Construction of bootstrapped correlation matrix S^c

for $d_a \in \{mRNA, CNA, miRNA, methylation, mutation\}$ **do**
for $d_b \in \{mRNA, CNA, miRNA, methylation, mutation\}$ **do**
$T_{d_a} = D_{d_a}$ ▷ temporary variables
$T_{d_b} = D_{d_b}$
Remove columns from T_{d_a} and T_{d_b} with patients that are not present in both datasets and ensure both matrices have the same column sorting.
Remove 10% random columns from T_{d_a} and T_{d_b}
$S_{ab} = corr(T'_{d_a}, T'_{d_b}, 'type', 'Pearson')'$ ▷ e.g. Matlab command corr, calculating ▷ cross-correlations between all variables
if $d_a = mutation$ or $d_b = mutation$ **then**
if $S^{ij}_{ab} = NaN$ **then** ▷ Case of gene not mutated in any patient in cancer c
$S^{ij}_{ab} \leftarrow 0$
end if
end if
end for
end for

$$S_c \leftarrow \begin{pmatrix} S_{mRNA,mRNA} & S_{mRNA,CNA} & S_{mRNA,miRNA} & S_{mRNA,meth.} & S_{mRNA,mut.} \\ S_{CNA,mRNA} & S_{CNA,CNA} & S_{CNA,miRNA} & S_{CNA,meth.} & S_{CNA,mut.} \\ S_{miRNA,mRNA} & S_{miRNA,CNA} & S_{miRNA,miRNA} & S_{miRNA,meth.} & S_{miRNA,mut.} \\ S_{meth.,mRNA} & S_{meth.,CNA} & S_{meth.,miRNA} & S_{meth.,meth.} & S_{meth.,mut.} \\ S_{mut.,mRNA} & S_{mut.,CNA} & S_{mut.,miRNA} & S_{mut.,meth.} & S_{mut.,mut.} \end{pmatrix}$$

1.3.3 Filter for variables with correlations that will not affect the final network.

See section 1.1.6 above.

---

for all rows $i \in S$ **do**
  if $i \in \{miRNA, mut\}$ **then**
    if $s_{ij} < 0.75 \cdot 0.7 \, \forall j$ **then**
      Remove row and column i from S
    else
      if $s_{ij} < 0.75 \cdot 0.7 \, \forall j \in \{miRNA, mut\}$ & $s_{ij} < 0.7 \, \forall j \in \{mRNA, CNA, meth\}$ **then**
        Remove row and column i from S
      end if
    end if
  end if
end for

---

	mrna	cna	mirna	methylation	mutation	clinical
GBM						
mrna	529					
cna	509	564				
mirna	482	472	491			
methylation	279	278	278	288		
mutation	148	151	147	63	157	
clinical	529	561	491	286	157	603
OV						
mrna	568					
cna	560	565				
mirna	564	561	570			
methylation	551	548	553	557		
mutation	466	460	466	452	467	
clinical	568	563	568	555	467	598
BRCA						
mrna	779					
cna	766	848				
mirna	449	489	498			
methylation	488	541	372	547		
mutation	723	759	435	465	772	
clinical	779	840	494	539	772	865

	mrna	cna	mirna	methylation	mutation	clinical
HNSC						
mrna	263					
cna	260	288				
mirna	227	250	252			
methylation	263	288	252	292		
mutation	261	286	251	290	306	
clinical	261	286	250	290	289	293
UCEC						
mrna	335					
cna	329	430				
mirna	197	245	249			
methylation	221	314	249	334		
mutation	241	243	110	134	248	
clinical	335	412	244	313	248	455
KIRC						
mrna	470					
cna	461	490				
mirna	198	209	213			
methylation	264	278	145	283		
mutation	270	292	141	199	293	
clinical	470	490	213	283	293	502

	mrna	cna	mirna	methylation	mutation	clinical
LUSC						
mrna	224					
cna	224	290				
mirna	132	137	137			
methylation	95	149	25	150		
mutation	178	178	110	74	178	
clinical	224	279	137	145	178	280
COAD						
mrna	192					
cna	184	414				
mirna	0	191	191			
methylation	30	254	191	255		
mutation	165	363	166	208	369	
clinical	192	414	191	255	369	424

Table S2: Number of patients for each combination of data types.

1.4 The Cancer Landscapes Resource

1.4.1 Scope of Cancer Landscapes

The Cancer Landscapes system is designed to be an accessible tool for analysis of multi-cancer models. In comparison to existing tools (Table below), it has a complementary set of features.

	**Cancer Landscapes**	**cBio portal**	**Cytoscape**	**Cancer Regulome**	**Cancer Genome Browser**
Web tool	Yes	Yes	Limited*	Yes	Yes
Pan-cancer networks	Yes	No	NA	No	NA
Single cancer models	Yes	Yes	NA	Yes	NA
Interactive visualization	Yes	Yes	Yes	Yes	Yes
Pathway analysis	Yes	No	Yes	No	No
Third party plugins	No	API	Yes	No	API
Cytoscape compatible	Yes	Yes	Yes	No	NA
TCGA Data download	No	Yes	NA	No	Yes

Table S3: Feature comparison of notable software with similar range of function as the Cancer Landscapes system. The comparison includes data portals (**cBio portal** (21; 22), **Cancer Genome Browser** (23)), pre made model visualization (**Cancer Regulome** (24)) and standard network tools (**Cytoscape** (25; 26)). NA denotes that the feature is not applicable in the context of that software. *Cytoscape Web is under development but not fully comparable to Cytoscape. Other software with some overlapping features are available, e.g; Integrative Genomics Viewer (27), **Circos** (28) and Oncomine(29).

1.4.2 Online application

The Cancer Landscapes (CL) browser has been implemented as a web resource for several reasons, the main one being accessibility. To facilitate the exploration of the models, we make the models and analysis results available through any modern web browser (Chrome, Firefox, Safari, Opera, IE 9+) and an internet connection. Although the system works well in most browsers on a reasonably modern computer, we recommend the use of Google Chrome, because it shows better performance across core technologies.

The Cancer Landscapes browser provides a large number of analysis tools that can be applied to any of the models available in the CL library. These tools can be divided into three categories; basic network exploration, integration of biological data and mathematical methods. We also allow the user to download results of their analysis, or the entire model, for further exploration in other software, such as Cytoscape.

1.4.3 Basic network exploration

A network model is drawn as a number of nodes representing variables and links (edges) between them representing associations. Each node has a specific data type (e.g. gene expression or methylation) and each link can be present in different data classes included in the model, e.g. different types of cancer. The user can choose which data types, which data classes and any combination of these to view. The user can also choose to view links that are present in all of the classes in the model, links unique to each cancer and any combination thereof. There are also multiple options for adjusting the appearance of the network, e.g. colors and link styles. The user can zoom (scroll) and pan (click and drag) around the network to closer inspect regions of interest. By clicking on a node, a window with further information about that node appears. Here the user can view the local properties of that node as well as access further information such as the entry for a gene in the NCBI or OMIM databases.

1.4.4 Links to third-party databases and underlying molecular data

Information about genes in the models, such as clinical results and chromosomal positions, is sourced from the NCBI, OMIM, PubMed, GOslim and PathwayCommons databases. This data is retrieved on-the-fly from each of these sources once the user requests information about a particular gene (by clicking the associated node in the network). This reduces the amount of data transferred and also guarantees that up-to-date data is always presented to the user.

The user can highlight genes from any pathway by searching through lists of the available pathways. It is also possible to manually select a region of the network and see which pathways that most overlap with nodes in that region. Such a region may be defined as an area within some distance of a certain point, or as a community within a certain number of steps in the network from a particular node. This allows the user to easily get a feel for how different biological functional categories are spatially distributed, as well as how they relate to other properties of the network structure, such as differences between classes. After performing a clustering of the network, as discussed below, each gene group is associated with the pathway with which it most overlaps. This gives an overview of which functional categories are present as contiguous network structures in different classes of the model.

For each gene (or any node associated to a gene) the user can view the corresponding text entry from NCBI and OMIM. These entries contain information about the recorded function of the gene and available knowledge pertaining to it. PubMed searches for the gene and the gene in combination with a number of informative terms are presented with the corresponding number of hits. The search terms include cancer terms representing specific cancers included in the model. This quickly gives the user an overview of how

well explored a particular gene is in relation to different diagnoses.

The network models presented in the system builds on a large amount of data from a wide range of diagnoses and data platforms. To fully appreciate the network models it is useful to have access to the underlying data. In the Cancer Landscapes web browser the user can view measured data for all nodes as well as survival data for patients included in any particular model. This information is presented as scatter plots between the underlying data corresponding to nodes in the network, i.e. the data from which the links are inferred. These plots illustrate the differential behavior between classes of data and allow the user to get the unfiltered view, not dependent on any network inference.

Kaplan-Meier survival curves with censoring can be viewed for groups of patients corresponding to the classes of the model. Patients can also be stratified based on the upper and lower quartile of data values corresponding to a node (e.g. mRNA expression). The corresponding Kaplan-Meier curves thus capture the difference in survival between groups of patients with differential node data measurements. For nodes corresponding to discrete data, a grouped boxplot is shown to illustrate the difference in values of one node when stratifying based on the discrete values of another node.

1.4.5 Scoring individual nodes by centrality and survival association

We provide the user with multiple measures of node importance to aid in the search for gene targets. Currently, there are three available measures of node importance or *node centrality*.

- **Survival Association.** The survival differences between patients stratified by the upper and lower quartile of data for each node (e.g. mRNA expression) is tested via the log-rank test and the corresponding p-value computed. The node-size is then presented as proportional to the $-log10(p\text{-value})$ to visually summarize the network structure in terms of survival association.
- **Node Degree.** The degree centrality of a node is simply the number of outgoing and incoming edges (links) to that node.
- **Betweenness centrality.** Betweenness centrality (30) corresponds to how often a node is found on the shortest path between two other nodes. To compute this, we solve a shortest path problem for all pairs of nodes and keep track of how often each node is found on any of the shortest paths. The resulting values are then scaled so that all node centrality values lie between zero and one. The all-pairs shortest path problem is efficiently solved using the algorithm proposed by Brandes (31).
- **PageRank.** The PageRank measure of node importance is one of the central parts of the Google system for search results ranking (32). This centrality measure corresponds to the average time spent

at a certain node when randomly browsing the web where nodes are webpages and links are hyperlinks between pages. The PageRank problem is an eigenvalue problem, which we solve by power iteration. This is done after decomposing the transition probability matrix into a sparse matrix and dense vectors. This allows the matrix multiplication in the power iteration step to be efficiently performed for sparse networks.

1.4.6 Network layout

The graphical display of the network representing a model is integral to the interpretation of that model. Network layout procedures come in many flavors and each has their benefits and drawbacks. To make the layout of networks flexible the system provides an initial layout that the user can modify.

The layout algorithm chosen is comprised of three steps. First, the network is split into connected components for which layout parameters can be set individually. The components are positioned using a circle pack layout and given room based on the number of nodes in the component. Then, the CNA-nodes of each component are, optionally, placed on a circle on the perimeter of that component. This option is included to give a biologically relevant structure to the position of network nodes. Finally, the nodes of each component are iteratively positioned using the ForceAtlas2 algorithm (33).

The ForceAtlas2 algorithm is similar to the commonly used force directed algorithm layout proposed by Fruchterman and Reingold (34) in which nodes attract each other with a linear force and repels each other with a force proportional to the square of the distance between them. ForceAtlas2 uses a linear attraction force but a repulsion that depends on the number of links adjacent to a node. The goal with this adjustment is to let poorly connected nodes be placed closer to highly connected ones. ForceAtlas2 also uses an adaptive scheme to adjust the distance a node is allowed to move in each iteration. This has the same effect as the cooling temperature in the force directed algorithm. These parameters can be chosen individually for each component in the system, which provides a great deal of adaptability to the algorithm.

1.4.7 Identification and enrichment analysis of network modules.

Clustering was performed using hierarchical clustering with average linkage. The distance matrix used was 1-topological overlap between nodes calculated from the joint graph of all cancers (1 if links exists in at least one cancer, otherwise 0).

Topological overlap (35) between nodes i and j is defined as: $TO(i,j) = \frac{|N(i) \cap N(j)| + a_{ij}}{min(|N(i)|, |N(j)|) + 1 - a_{ij}}$, where

$N(i)$ denotes the neighborhood of node i, $||$ denotes the cardinality and a_{ij} is an element in the adjacency matrix.

The total graph was split into connected components and the clustering performed on each component individually to save computation time (since disconnected components do not cluster and the clustering algorithm has non-linear time complexity). The number of clusters (for each component) was chosen based in the average silhouette width for the cluster assignment obtained by cutting the dendrogram into a fixed number of clusters k (cluster(.,'maxclust ',k) in Matlab 2014b). Clusters with less than 10 nodes were discarded. The rule for choosing k was as follows; (1) choose the largest feasible k, (2) check if there is any k_{new} s.t. the average silhouette width of the assignment given by k_{new} is at least 95% of that for k, (3) set k = k_{new} and repeat until no new k can be found.

1.4.8 Characterization of network modules.

Enrichment of a certain feature across clusters was calculated for a number of features (e.g. survival associated nodes for each cancer, pathways), by a hypergeometric test (Fisher's exact test, calculated - using MATLAB notation - as 1-hygecdf(x-1,M,K,N), where M is the number of network nodes, K the number of labeled nodes, K the size of the cluster and x is the number of labeled nodes in the cluster). A node was defined as labeled when the corresponding gene is annotated with the feature of interest, e.g. membership in a certain pathway.

The *p*-values were adjusted using Benjamini-Hochberg correction, for each feature separately and considered significant if they were less than 0.05 after this. To define survival associated nodes for this analysis, we used a Kaplan-Meier log-rank *p*-value cutoff of 0.05 (this is a deliberately inclusive threshold to avoid very low counts in enrichment testing).

For completeness, a full list of network modules analyzed in the paper is found in a supplementary data file. In general, however, we recommend using the Cancer Landscapes system for exploring modules.

1.5 Analysis of co-occurrence between *IDH1* mutation and chromosome 11p

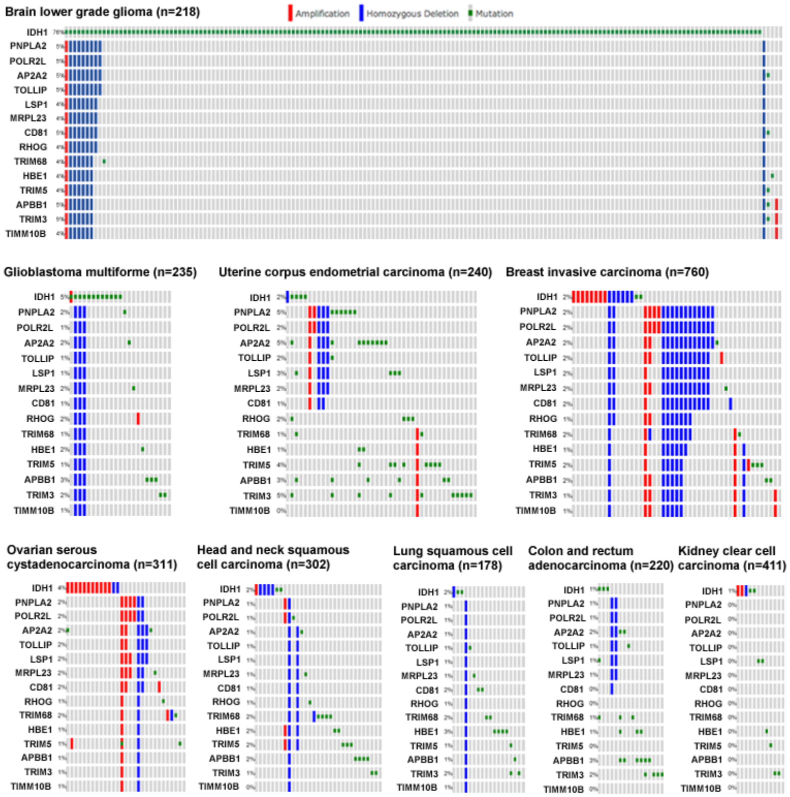

Figure S9: 11p15 deletion co-occurs with *IDH1* mutation (a) Oncoprint plot (22) illustrating co-occurrence of *IDH1* mutations and homozygous deletions in 14 genes located in the 11p15 region found to be correlated by our model in the eight cancers analyzed. All high-grade glioma patients carrying homozygous deletions in these genes have *IDH1* mutations (p<0.00001, see main paper). While the co-occurrence is present in the majority of low-grade glioma patients with both types of mutations (elevated odds ratio, but not significant), it is not observed in the other types of cancers.

References

[1] Witten, D. M., Friedman, J. H., and Simon, N. (2011) New insights and faster computations for the graphical lasso. *Journal of Computational and Graphical Statistics,* **20**(4), 892–900.

[2] Zou, H. and Hastie, T. (2005) Regularization and variable selection via the elastic net. *Journal of the Royal Statistical Society: Series B (Statistical Methodology),* **67**(2), 301–320.

[3] Zhuang, J., Widschwendter, M., and Teschendorff, A. E. (2012) A comparison of feature selection and classification methods in DNA methylation studies using the Illumina Infinium platform. *BMC bioinformatics,* **13**(1), 59.

[4] Waldmann, P., Mészáros, G., Gredler, B., Fuerst, C., and Sölkner, J. (2013) Evaluation of the lasso and the elastic net in genome-wide association studies. *Frontiers in genetics,* **4**.

[5] Hoefling, H. (January, 2010) A Path Algorithm for the Fused Lasso Signal Approximator. *dx.doi.org,* **19**(4), 984–1006.

[6] Danaher, P., Wang, P., and Witten, D. M. (March, 2014) The joint graphical lasso for inverse covariance estimation across multiple classes. *Journal of the Royal Statistical Society: Series B (Statistical Methodology),* **76**(2), 373–397.

[7] Edwards, D. Introduction to graphical modelling. (1995).

[8] Banerjee, O., El Ghaoui, L., and d'Aspremont, A. (June, 2008) Model Selection Through Sparse Maximum Likelihood Estimation for Multivariate Gaussian or Binary Data. *The Journal of Machine Learning Research,* **9**, 485–516.

[9] Liu, H., Han, F., Yuan, M., Lafferty, J., and Wasserman, L. (2012) High-dimensional semiparametric Gaussian copula graphical models. *The Annals of Statistics,* **40**(4), 2293–2326.

[10] John, B., Enright, A. J., Aravin, A., Tuschl, T., Sander, C., and Marks, D. S. (2004) Human microRNA targets. *PLoS biology,* **2**(11), e363.

[11] Griffiths-Jones, S., Saini, H. K., van Dongen, S., and Enright, A. J. (2008) miRBase: tools for microRNA genomics. *Nucleic acids research,* **36**(suppl 1), D154–D158.

[12] Zou, H. (2006) The adaptive lasso and its oracle properties. *Journal of the American statistical association,* **101**(476), 1418–1429.

[13] Jörnsten, R., Abenius, T., Kling, T., Schmidt, L., Johansson, E., Nordling, T. E. M., Nordlander, B., Sander, C., Gennemark, P., Funa, K., Nilsson, B., Lindahl, L., and Nelander, S. (April, 2011) Network modeling of the transcriptional effects of copy number aberrations in glioblastoma.. *Molecular systems biology,* **7**(1), 486–486.

[14] Li, S., Hsu, L., Peng, J., and Wang, P. (2013) Bootstrap inference for network construction with an application to a breast cancer microarray study. *The annals of applied statistics,* **7**(1), 391.

[15] Sanchez, J., Jauhiainen, A., Nelander, S., and Jornsten, R. (2014) Network sparsity selection and robust estimation via bootstrap with applications to genomic data.. *Manuscript included in PhD thesis,Chalmers Institute of Technology,* **.**(Dec.2014), .

[16] Boyd, S., Parikh, N., Chu, E., Peleato, B., and Eckstein, J. (2011) Distributed optimization and statistical learning via the alternating direction method of multipliers. *Foundations and Trends® in Machine Learning,* **3**(1), 1–122.

[17] Ye, G.-B. and Xie, X. (April, 2011) Split Bregman method for large scale fused Lasso. *Computational Statistics & Data Analysis,* **55**(4), 1552–1569.

[18] Langfelder, P. and Horvath, S. (2008) WGCNA: an R package for weighted correlation network analysis. *BMC Bioinformatics,* **9**(1), 559.

[19] Margolin, A. A., Nemenman, I., Basso, K., Wiggins, C., Stolovitzky, G., Favera, R. D., and Califano, A. (2006) ARACNE: an algorithm for the reconstruction of gene regulatory networks in a mammalian cellular context. *BMC Bioinformatics,* **7**(Suppl 1), S7.

[20] Reshef, D. N., Reshef, Y. A., Finucane, H. K., Grossman, S. R., McVean, G., Turnbaugh, P. J., Lander, E. S., Mitzenmacher, M., and Sabeti, P. C. (2011) Detecting novel associations in large data sets. *Science,* **334**(6062), 1518–1524.

[21] Cerami, E., Gao, J., Dogrusoz, U., Gross, B. E., Sumer, S. O., Aksoy, B. A., Jacobsen, A., Byrne, C. J., Heuer, M. L., Larsson, E., Antipin, Y., Reva, B., Goldberg, A. P., Sander, C., and Schultz, N. (2012) The cBio cancer genomics portal: an open platform for exploring multidimensional cancer genomics data. *Cancer Discov,* **2**(5), 401–404.

[22] Gao, J., Aksoy, B. A., Dogrusoz, U., Dresdner, G., Gross, B., Sumer, S. O., Sun, Y., Jacobsen, A., Sinha, R., Larsson, E., Cerami, E., Sander, C., and Schultz, N. (2013) Integrative Analysis of Complex Cancer Genomics and Clinical Profiles Using the cBioPortal. *Sci Signal,* **6**(269), pl1.

[23] Cline, M. S., Craft, B., Swatloski, T., Goldman, M., Ma, S., Haussler, D., and Zhu, J. (2013) Exploring TCGA Pan-Cancer data at the UCSC Cancer Genomics Browser.. *Scientific reports,* **3**, 2652.

[24] The Cancer Genome Atlas Research Network (2012) Comprehensive molecular characterization of human colon and rectal cancer. *Nature,* **487**(7407), 330–337.

[25] Shannon, P., Markiel, A., Ozier, O., Baliga, N. S., Wang, J. T., Ramage, D., Amin, N., Schwikowski, B., and Ideker, T. (2003) Cytoscape: a software environment for integrated models of biomolecular interaction networks. *Genome research,* **13**(11), 2498–2504.

[26] Cline, M. S., Smoot, M., Cerami, E., Kuchinsky, A., Landys, N., Workman, C., Christmas, R., Avila-Campilo, I., Creech, M., Gross, B., Hanspers, K., Isserlin, R., Kelley, R., Killcoyne, S., Lotia, S., Maere, S., Morris, J., Ono, K., Pavlovic, V., Pico, A. R., Vailaya, A., Wang, P. L., Adler, A., Conklin, B. R., Hood, L., Kuiper, M., Sander, C., Schmulevich, I., Schwikowski, B., Warner, G. J., Ideker, T., and Bader, G. D. (2007) Integration of biological networks and gene expression data using Cytoscape. *Nat Protoc,* **2**(10), 2366–2382.

[27] Thorvaldsdottir, H., Robinson, J. T., and Mesirov, J. P. (2013) Integrative Genomics Viewer (IGV): high-performance genomics data visualization and exploration. *Brief Bioinform,* **14**(2), 178–192.

[28] Krzywinski, M., Schein, J., Birol, I., Connors, J., Gascoyne, R., Horsman, D., Jones, S. J., and Marra, M. A. (2009) Circos: an information aesthetic for comparative genomics. *Genome research,* **19**(9), 1639–1645.

[29] Rhodes, D. R., Kalyana-Sundaram, S., Mahavisno, V., Varambally, R., Yu, J., Briggs, B. B., Barrette, T. R., Anstet, M. J., Kincead-Beal, C., Kulkarni, P., Varambally, S., Ghosh, D., and Chinnaiyan, A. M. (2007) Oncomine 3.0: genes, pathways, and networks in a collection of 18,000 cancer gene expression profiles. *Neoplasia,* **9**(2), 166–180.

[30] Freeman, L. C. (March, 1977) A Set of Measures of Centrality Based on Betweenness. *Sociometry,* **40**(1), 35.

[31] Brandes, U. (August, 2010) A faster algorithm for betweenness centrality*. *dx.doi.org,* **25**(2), 163–177.

[32] Page, L., Brin, S., Motwani, R., and Winograd, T. (November, 1999) The PageRank Citation Ranking: Bringing Order to the Web.. *stanford infolab technical report,* **1999-66**.

[33] Jacomy, M., Heymann, S., Venturini, T., and Bastian, M. (2011) Forceatlas2, a graph layout algorithm for handy network visualization, ... http://www. medialab. sciences-po. fr/ ..., .

[34] Fruchterman, T. M. J. and Reingold, E. M. (November, 1991) Graph drawing by force-directed placement. *Software: Practice and Experience,* **21**(11), 1129–1164.

[35] Ravasz, E., Somera, A. L., Mongru, D. A., Oltvai, Z. N., and Barabási, A.-L. (2002) Hierarchical organization of modularity in metabolic networks. *science,* **297**(5586), 1551–1555.
